# Chromosome fusions repatterned recombination rate and facilitated reproductive isolation during *Pristionchus* nematode speciation

**DOI:** 10.1038/s41559-022-01980-z

**Published:** 2023-01-30

**Authors:** Kohta Yoshida, Christian Rödelsperger, Waltraud Röseler, Metta Riebesell, Simo Sun, Taisei Kikuchi, Ralf J. Sommer

**Affiliations:** 1 Department for Integrative Evolutionary Biology, Max Planck Institute for Biology, Tübingen, Germany; 2 Faculty of Medicine, University of Miyazaki, Miyazaki, Japan

**Keywords:** Speciation, Zoology

## Abstract

Large-scale genome-structural evolution is common in various organisms. Recent developments in speciation genomics revealed the importance of inversions, whereas the role of other genome-structural rearrangements, including chromosome fusions, have not been well characterized. We study genomic divergence and reproductive isolation of closely related nematodes: the androdioecious (hermaphroditic) model *Pristionchus pacificus* and its dioecious sister species *Pristionchus exspectatus*. A chromosome-level genome assembly of *P. exspectatus* using single-molecule and Hi-C sequencing revealed a chromosome-wide rearrangement relative to *P. pacificus*. Strikingly, genomic characterization and cytogenetic studies including outgroup species *Pristionchus occultus* indicated two independent fusions involving the same chromosome, ChrIR, between these related species. Genetic linkage analysis indicated that these fusions altered the chromosome-wide pattern of recombination, resulting in large low-recombination regions that probably facilitated the coevolution between some of the ~14.8% of genes across the entire genomes. Quantitative trait locus analyses for hybrid sterility in all three sexes revealed that major quantitative trait loci mapped to the fused chromosome ChrIR. While abnormal chromosome segregations of the fused chromosome partially explain hybrid female sterility, hybrid-specific recombination that breaks linkage of genes in the low-recombination region was associated with hybrid male sterility. Thus, recent chromosome fusions repatterned recombination rate and drove reproductive isolation during *Pristionchus* speciation.

## Abstract (web summary)

Genomic analyses show that two independent fusions involving the same chromosome altered recombination patterns and contributed to reproductive isolation between two *Pristionchus* nematodes.

## Main

Chromosome fusions are one of the major types of mutations shaping the evolution and divergence of genomes in different species, and meta-analyses of changes in chromosome numbers have suggested their importance in speciation in mammals, reptiles and butterflies^1–4^. Early studies proposed that reproductive isolation is caused by abnormal chromosomal segregation in hybrid meiosis^5,6^. However, experimental evidence for such meiotic abnormalities is not always found^7,8^. Instead, it has been proposed that chromosome rearrangements reduce gene flow by the alteration of recombination patterns^7,9^. Chromosomal rearrangements often suppress recombination within or around the respective region in hybrids between populations fixed for alternative arrangements, which can increase genome-sequence divergence between species^10^, protect species-specific gene combinations from introgression^7^ and facilitate the evolution of reproductive isolation^11^. Indeed, chromosomal inversions restrict recombination and gene flow between species and promote divergence of genes between species^12–16^, and their role in reproductive isolation was empirically confirmed in several organisms^17–20^. By contrast, the effect of recombination reduction by chromosome fusions was only theoretically proposed^21,22^ but not experimentally confirmed. Therefore, direct evidence for reproductive isolation by chromosome fusions has been limited, and its evolutionary mechanism remains elusive.

In this Article, we addressed the role of chromosome fusions in reproductive isolation in nematodes, which form one of the most diverse phyla in the animal kingdom with important ecosystem functions^23,24^. Given their small body size and the scarcity of distinctive morphological features, the systematic status of many nematode taxa is difficult to determine^25^. As a result, the mechanisms associated with nematode speciation are little understood, and current knowledge is restricted largely to the genus *Caenorhabditis*^26–28^. However, detailed sampling has been initiated in various nematode groups, resulting in extensive lists of closely related species in some taxa.

**Fig. 1:**
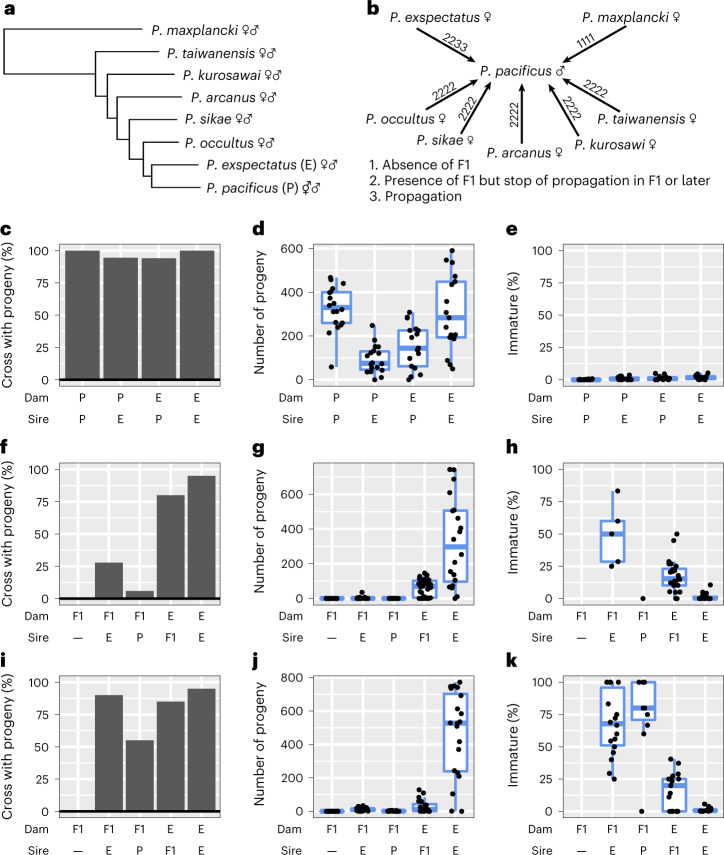
Reproductive isolation between *P. pacificus* and *P. exspectatus*. **a**, Phylogeny of the *pacificus* complex with the outgroup species *P. maxplancki*. The tree is a subtree of the reported phylogeny based on phylotranscriptomics^38^. The reproductive mode of each species is shown as symbol. ⚥♂, androdioecy; ♀♂, dioecy. **b**, Crossing experiments between *P. pacificus* male and females of related species. The species of the *pacificus* complex and *P. maxplancki* were crossed with *P. pacificus*. The four numbers at the arrows indicate the results of four-replicate crosses of three females and six males. The presence/absence of F1 progeny was first tested. If F1 were produced, F1 siblings were allowed to mate with each other, and their ability to propagate in one month (~six generations) was tested. **c**–**k**, Results of quantitative reproduction tests of hybrid crosses between *P. pacificus* and *P. exspectatus* (**c**–**e**), backcrosses with F1 animals produced by a cross between a *P. exspectatus* female and a *P. pacificus* male (**f**–**h**), backcrosses with F1 animals produced by a cross between a *P. pacificus* hermaphrodite and a *P. exspectatus* male (**i**–**k**). The proportion of crosses with progeny (**c**,**f**,**i**), the number of the progeny (**d**,**g**,**j**) and the proportion of immature progeny (**e**,**h**,**k**) are compared between different crosses. The first column of the backcrosses is the result for F1 females without males to test fertility in selfing. P and E indicate *P. pacificus* and *P. exspectatus*, respectively. For the number of progeny and proportion of immature progeny, all replicates are shown as jitter plots while the box plot (upper whisker, the largest data point less than the third quartile + 1.5 × interquartile range; upper bound, the third quartile; centre line, median; lower bound, the first quartile; lower whisker, the smallest data point more than the first quartile − 1.5 × interquartile range) are shown when at least five data points exist. Old dams (four days after J4 stage) were used for the hybrid cross whereas young dams (J4 stage) were used for the backcross. The sample number is shown in Supplementary Table 1.

One such group is *Pristionchus* nematodes related to the model species *P. pacificus*^29^. These soil nematodes are often found in association with scarab beetles, with sampling efforts around the world resulting in the description of 49 culturable species^29^. These species have been distinguished by differences in morphology and genetic divergence (from RNA sequencing) and on the basis of reproductive-isolation experiments. Interestingly, *P. pacificus* and its closest relatives can form F1 hybrids and comprise the ‘*pacificus* complex’, representing some of the most closely related species described in nematodes (Fig. 1a)^30,31^. While the estimation of divergence in nematodes is complicated by the scarceness of fossils, estimates based on synonymous substitution rates suggest that the most closely related species pair has diverged 12–28 million generations ago^32,33^.

*P. pacificus* is an androdioecious species that reproduces by self-fertilization of hermaphrodites or outcrossing of hermaphrodites to males^34^ and provides the same experimental advantages as *C. elegans*, such as a short generation time of 3–4 days, small genome size (~160 megabases (Mb)) (ref. ^35^) and amenability to forward and reverse genetic manipulation, including CRISPR/Cas9 engineering^36,37^. Importantly, hermaphroditic species, such as *P. pacificus*, are phylogenetically most closely related to dioecious species^38,39^. Indeed, the *pacificus* complex includes *P. pacificus* and six dioecious species, with *P. exspectatus* representing the molecular ‘sister species’ of *P. pacificus* (Fig. 1a).

In the present study, we first characterize the reproductive isolation in the *pacificus*-complex and analyse the genomes of the most closely related species. We found multiple chromosome fusions among the species and investigated a role of chromosome fusions in speciation by quantitative trait locus (QTL) analyses and subsequent genetic analyses.

## Varied degree of hybrid sterility in the *pacificus* complex

To compare the status of reproductive isolation between the hermaphrodite *P. pacificus* and other species of the *pacificus* complex, we crossed *P. pacificus* males with females of seven related species and investigated the resulting hybrids in intercrosses using a method previously reported^31^ (Fig. 1b). We confirmed previous findings^31^ that F1 animals were produced by crossing *P. pacificus* males with females of all species in the *pacificus* complex but not by crossing them with females of the outgroup *P. maxplancki* (Fig. 1b). However, these hybrid animals did not sustainably propagate, except for *P. exspectatus*. In two of four crosses between *P. pacificus* and the most closely related species, *P. exspectatus*, we observed sustainable propagation with continuous production of newborn larvae for at least one month (~6 generations).

**Extended Data Fig. 1:**
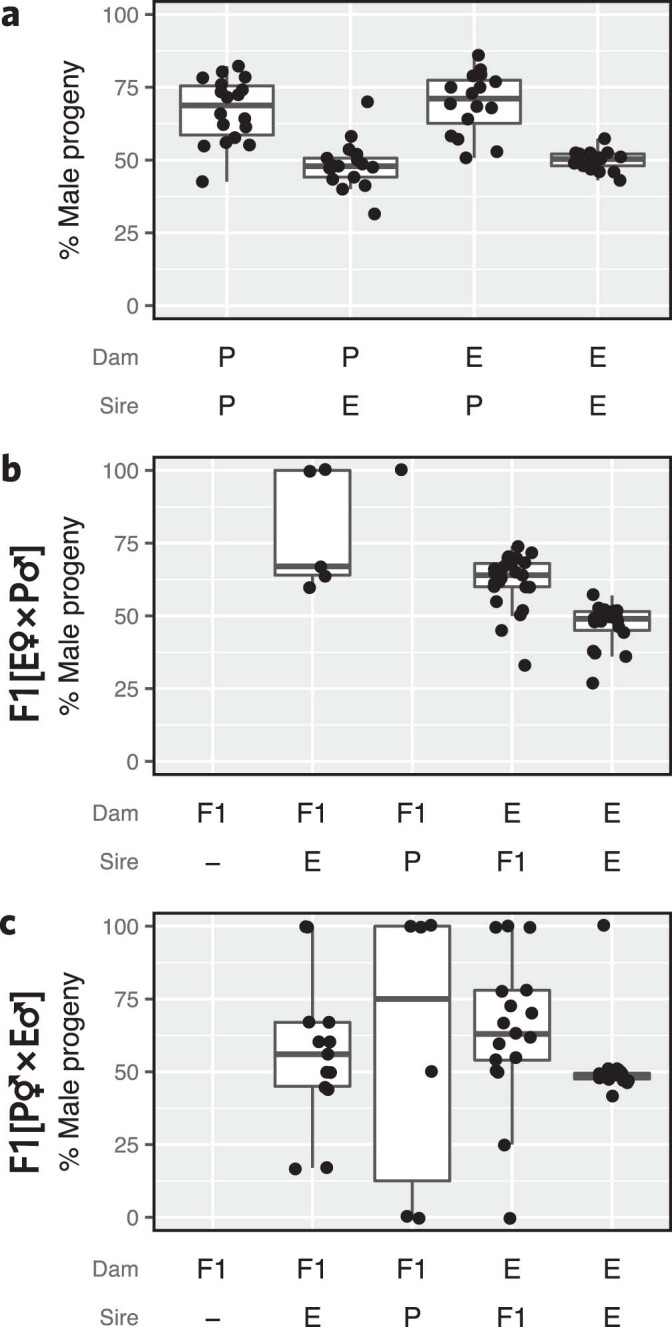
Hybrid crosses between *P. exspectatus* and *P. pacificus*. Sex ratio of progeny of hybrid crosses of *P. exspectatus* and *P. pacificus* (**a**) and backcrosses of F1 hybrids produced by crosses between *P. exspectatus* females and *P. pacificus* males (**b**) and backcrosses of F1 hybrids produced by crosses between *P. pacificus* hermaphrodites and *P. exspectatus* males (**c**). The proportion of males among the mature progeny was calculated. The first column of the backcrosses is the result for F1 females without males to test their selfing fertility. P and E indicate *P. pacificus* and *P. exspectatus*, respectively. All replicates are shown as jitter plots while the box plots (upper whisker, the largest data point less than the third quartile+1.5×interquartile range; upper bound, the third quartile; centre line, median; lower bound, the first quartile; lower whisker, the smallest data point more than the first quartile−1.5×interquartile range) are shown when at least 5 data points exist. The sample number is shown in Supplementary Table 1.

To test the reproductive status of hybridization between *P. pacificus* and *P. exspectatus* in a quantitative manner, we analysed the number of progeny and the sex ratio in hybrid crosses between *P. pacificus* and *P. exspectatus* and compared these values with those of intraspecific crosses (Fig. 1c–e and Extended Data Fig. 1a; sample number shown in Supplementary Table 1). Old females or hermaphrodites that have run out of self-sperm were used in this experiment (Methods). Almost all crosses produced progeny, suggesting no prezygotic isolation (Fig. 1c). However, the number of progeny was lower in hybrid crosses (asymptotic Wilcoxon–Mann–Whitney test; any pairs between interspecies and intraspecies crosses, *Z* > 2.44; two-tailed *P* < 0.015; Fig. 1d). Only a small number of animals in hybrid crosses experienced developmental failure and remained immature (the mean ± standard error of proportion of immature progeny of the crosses: *P. pacificus* × *P. pacificu*s, 0.0017 ± 0.0006; *P. pacificus* × *P. exspectatus*, 0.0098 ± 0.0028; *P. exspectatus* × *P. pacificus*, 0.0128 ± 0.0039; *P. exspectatus* × *P. exspectatus*, 0.0185 ± 0.0039; Fig. 1e). The sex ratio of progeny was different between crosses, reflecting the different sire species (the mean ± standard error of male ratio of the progeny of the crosses: *P. pacificus* × *P. pacificu*s, 0.670 ± 0.026; *P. pacificus* × *P. exspectatus*, 0.483 ± 0.020; *P. exspectatus* × *P. pacificus*, 0.696 ± 0.026; *P. exspectatus* × *P. exspectatus*, 0.498 ± 0.008; Extended Data Fig. 1a).

Next, we used backcrossing of F1 animals produced by crosses between *P. exspectatus* females and *P. pacificus* males (F1[E × P]) to test the fertility of the F1 animals (Fig. 1f–h; sample number shown in Supplementary Table 1). We found that 6% and 28% of F1[E × P] females produced progeny when outcrossed with males of *P. pacificu*s and *P. exspectatu*s, respectively, whereas such females did not form any progeny without males (Fig. 1f). By contrast, 80% of F1[E × P] hybrid males (*N* = 30) produced progeny with females of *P. exspectatus*. Note that the numbers of progeny produced by these hybrid males is still lower than the progeny resulting from mating within *P. exspectatus* (asymptotic Wilcoxon–Mann–Whitney test; *Z* = 3.92, two-tailed *P* = 8.98 × 10^−5^; Fig. 1g) although this might reflect F1 hybrid male sterility or BC1 hybrid inviability. These results suggest different extents of hybrid sterility among sexes. First, F1[E × P] hybrid hermaphrodite sterility, that is, sterility in selfing, is complete. Second, F1[E × P] hybrid female sterility is severe but not complete, and third, F1[E × P] hybrid male sterility is at most partial. We also tested backcrosses of F1 produced by the reciprocal cross (F1[P × E]) and confirmed a similar trend (Fig. 1i–k; sample number shown in Supplementary Table 1). Note that the number of progeny produced by backcrosses of F1[P × E] females with *P. exspectatus* is larger than that of F1[E × P] females (Fig. 1i; asymptotic Wilcoxon–Mann–Whitney test: *Z* = 3.52, two-tailed *P* = 4.34 × 10^−4^) and not different from that of F1[P × E] males (Fig. 1j; asymptotic Wilcoxon–Mann–Whitney test, *Z* = 0.434, two-tailed *P* = 0.66). However, the F1[P × E] female produced a significantly higher percentage of immature progeny than did the F1[P × E] male (Fig. 1k; asymptotic Wilcoxon–Mann–Whitney test, *Z* = 4.79, two-tailed *P* = 1.66 × 10^−6^). Next, we tested intercrosses of F1 animals of both reciprocal crosses, which produced only few progeny (Supplementary Fig. 1; sample number shown in Supplementary Table 1). Finally, we also tested the reproductive capability of F1 hybrid males between *P. pacificus* and the other species (*N* = 25 each) and confirmed that hybrid males were reproductively functional only when closely related species were crossed (Supplementary Fig. 2). Together, our findings indicate that *P. exspectatus* and *P. pacificus* represent the most closely related species with partial fertility of hybrids.

## Comparative genomics of *P. exspectatus* and *P. pacificus*

**Extended Data Fig. 2:**
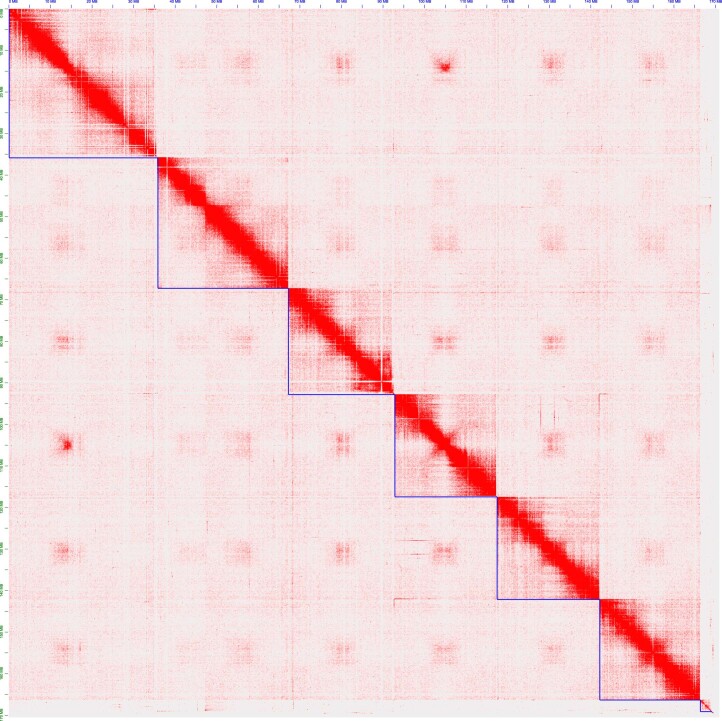
Contact map of Hi-C analysis of *P. exspectatus*. The contact map was drawn with 3D *de novo* assembly. The red plot indicates the contact detected in Hi-C analysis between the genomic positions. Blue lines indicate the boundaries of scaffolds. Scaffolds were ordered by size.

To analyse potential structural differences between the genomes of *P. pacificus* and *P. exspectatus*, we generated a chromosomal-level de novo assembly in *P. exspectatus*. First, we sequenced the *P. exspectatus* genome using the PacBio platform (Pacific Biosciences) and removed duplicates likely resulting from the remaining heterozygosity after inbreeding using Haplomerger2^40^. Next, we conducted Hi-C analysis to scaffold the assembly, resulting in six long chromosomes that contained 98.1% of genome sequences (Extended Data Fig. 2). This is an improvement relative to the previous fragmented version of *P. exspectatus* genome^33^ (Supplementary Table 2). The genome size of the new assembly is 170 Mb and is still longer than the 158 Mb genome of *P. pacificus*. We predicted a total of 31,021 genes in the *P. exspectatus* genome that contain 90% of benchmarking universal single-copy orthologues (BUSCO) as complete single-copy genes, whereas 28,896 genes were predicted in the *P. pacificus* genome (Supplementary Table 2). These differences are consistent with genome shrinkage and gene loss in androdioecious species as previously proposed for *Pristionchus* and *Caenorhabditis* nematodes^38,41^.

**Fig. 2:**
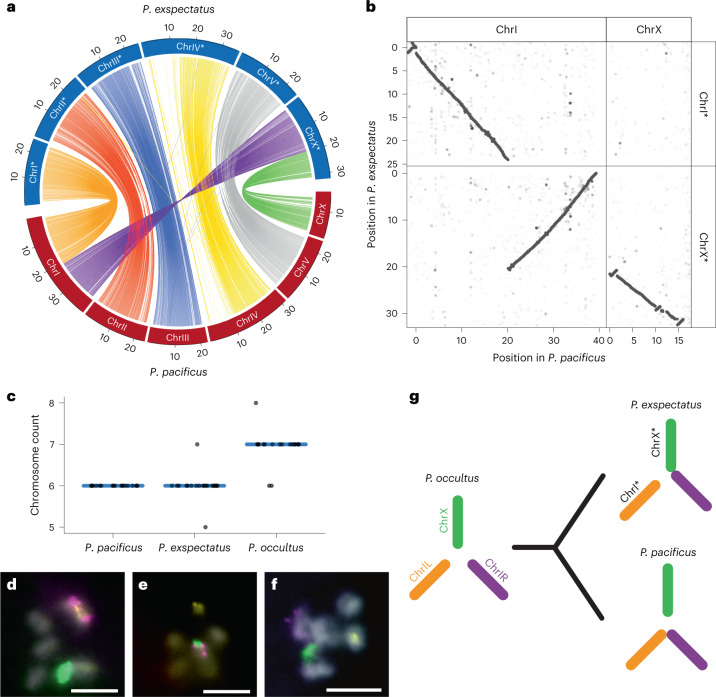
De novo assembly of *P. exspectatus* genome and karyotype analysis indicate two independent chromosome fusion events. **a**, Circos plot of nucleotide-based homologous loci between *P. pacificus* and *P. exspectatus*. The 100 kb sliding windows with more than 10% homologous sequence are shown as links. The colours reflect chromosomal regions in *P. pacificus*. The ChrIL and ChrIR (left and right of chromosome I 21.05 Mb, respectively) are shown in different colours (orange and purple, respectively). **b**, Dot plot of nucleotide-based homologous loci of chromosomes involved in the large interchromosomal rearrangement. Homologous single nucleotides downsized by 1/100 are plotted. **c**, Chromosome count of spermatocyte prophase I of three species. *P. pacificus*, *N* = 19; *P. exspectatus*, *N* = 21; *P. occultus*, *N* = 19. The mode is shown as a blue line. **d**–**f**, FISH mapping of prophase I spermatocyte of *P. pacificus* (**d**), *P. exspectatus* (**e**) and *P. occultus* (**f**) with the specific 21-mer of the three elements: ChrIL (yellow), ChrIR (magenta) and ChrX (green). 4,6-Diamidino-2-phenylindole (DAPI) staining is shown as grey. The scale bars are 5 µm. **g**, Karyotype evolution between the three investigated species.

**Extended Data Fig. 3:**
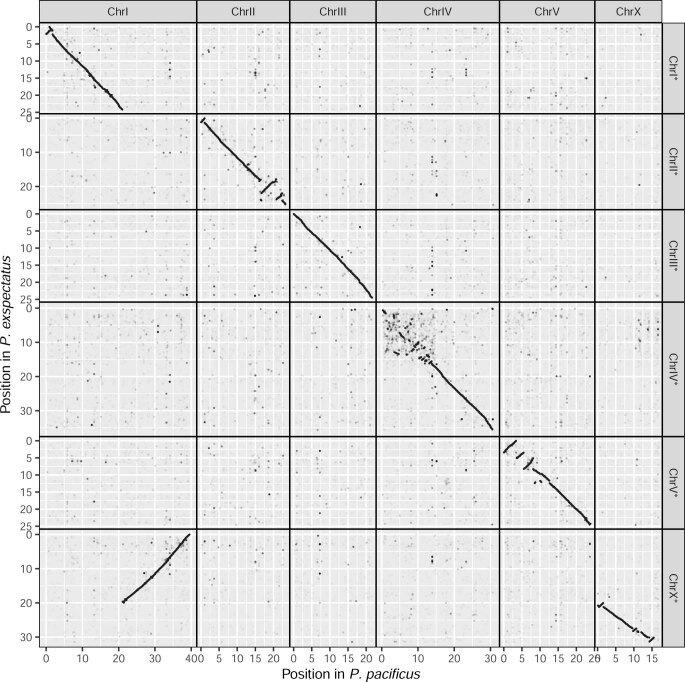
Dot plot of nucleotide-based comparative synteny across the genomes of *P. pacificus* and *P. exspectatus*. Homologous single nucleotides downsized by 1/100 were plotted.

**Extended Data Fig. 4:**
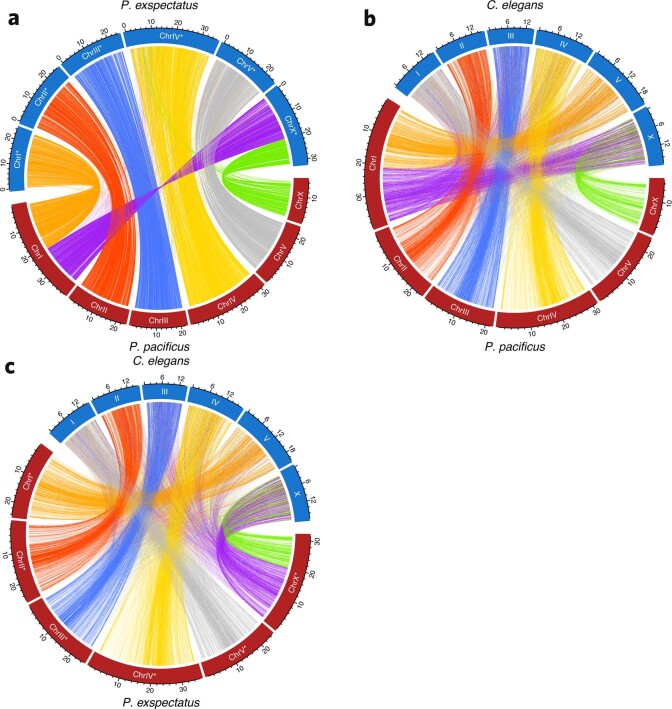
Circos plot of protein-based homologous loci. **a**, *P. pacificus vs. P. exspectatus*. **b**, *P. pacificus vs. C. elegans*. **c**, *P. exspectatus vs. C. elegans*. One-to-one orthologs were identified based on protein sequence homology. Position of one-to-one orthologs in two genomic sequences are shown as links. The colors of the links indicate different chromosomal regions in *P. pacificus* (**a, b**) or *P. exspectatus* (**c**). The genomic regions ChrIL, ChrIR, and ChrX (left and right of 21.05 Mb of ChrI and ChrX of *P. pacificus* or ChrI* and left and right of 20.00 Mb of ChrX* of *P. exspectatus*, respectively) are shown in different colors (orange, purple and green, respectively).

Next, we conducted comparative synteny analysis using the high-quality genome of *P. pacificus*^36^ on the basis of nucleotide-sequence (Fig. 2a,b and Extended Data Fig. 3) and protein-sequence homology (Extended Data Fig. 4a). Surprisingly, we found one large-scale interchromosomal rearrangement between the two species. Specifically, chromosome I (ChrI) of *P. pacificus* can be divided into two fragments, the left region (ChrIL) autosomal in both species and the right chromosomal region of 21.05 Mb (ChrIR) that is fused to the X chromosome in *P. exspectatus* (Fig. 2a,b). As a result, *P. pacificus* ChrI is substantially larger than all other chromosomes, whereas the X chromosome is the largest in *P. exspectatus*. To properly distinguish between the chromosomes, we use a star (*) when referring to the *P. exspectatus* patterns (for example, ChrX*; Fig. 2a). Thus, the two most closely related species in the *pacificus* complex are genetically differentiated through a large-scale interchromosomal rearrangement. This finding is in contrast to species within the genus *Caenorhabditis* that have many intrachromosomal, but no large interchromosomal, rearrangements^42,43^.

We also identified several intrachromosomal rearrangements larger than 100 kb using dot-plot analysis (Supplementary Fig. 3). For example, we found a large highly structured region on the left arm of Chromosome IV that is detected only by dot-plot analysis (Extended Data Fig. 3) because each homologous sequence fragment is too small to be detected in circos plots (Fig. 2a). The large inversions are distributed across different chromosomes and are often observed around the end of a chromosome (Supplementary Fig. 3).

## Two independent chromosome fusions during speciation

**Extended Data Fig. 5:**
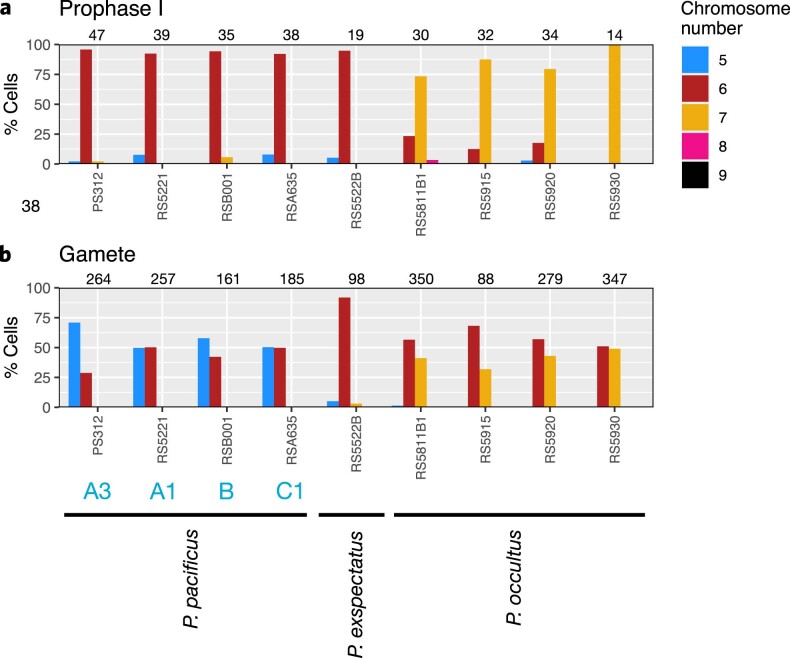
Karyotype analysis. The percentage of Prophase I (**a**) or gamete (**b**) cells with the number of chromosomes are shown in *Y*-axis. *X*-axis indicates strain ID. The count of cells from at least 9 individuals of each strain were summed to calculate the proportion. The number of cells is shown on top of each bar graph. Blue letters in *X*-axis indicates previously-reported clades of *P. pacificus*^36^. Species names are shown at the bottom. Variable and stable chromosome numbers in the male gamete (*i. e*. spermatozoa or sperm) are consistent with an XO system in *P. pacificus* and *P. occultus*, and an XY system in *P. exspectatus*.

To clarify the evolutionary context of the interchromosomal rearrangement, we conducted comparative cytogenetic analysis of the species, including the closest outgroup, *P. occultu*s. The observed chromosome number of *P. pacificus* and *P. exspectatus* is six as expected, whereas that of the outgroup species *P. occultus* is seven (Fig. 2c and Extended Data Fig. 5). This finding would be consistent with the possibility of two independent fusions of two of the three elements corresponding to *P. pacificus* ChrIL, ChrIR and ChrX in both lineages (Fig. 2g). Indeed, fluorescence in situ hybridization (FISH) analysis using probes designed for specific 21-mer sequences of the three elements supported two independent chromosome fusions. First, these three genetic markers are located on three different chromosomes in *P. occultus* (Fig. 2f). Second, markers for ChrIL and ChrIR are located on one chromosome in *P. pacificus* (Fig. 2d). And finally, markers for ChrIR and ChrX are located on the same chromosome in *P. exspectatus* (Fig. 2e). Karyotype analysis of different strains within species indicates stable karyotypes (Extended Data Fig. 5), although it is unclear whether the fusion in *P. exspectatus* is fixed in this species because only one isolate of *P. exspectatus* is available. Thus, two independent chromosome fusions involving the same chromosome, ChrIR, occurred in *P. pacificus* and *P. exspectatus*, and at least one of the fusions is fixed (Fig. 2g).

**Fig. 3:**
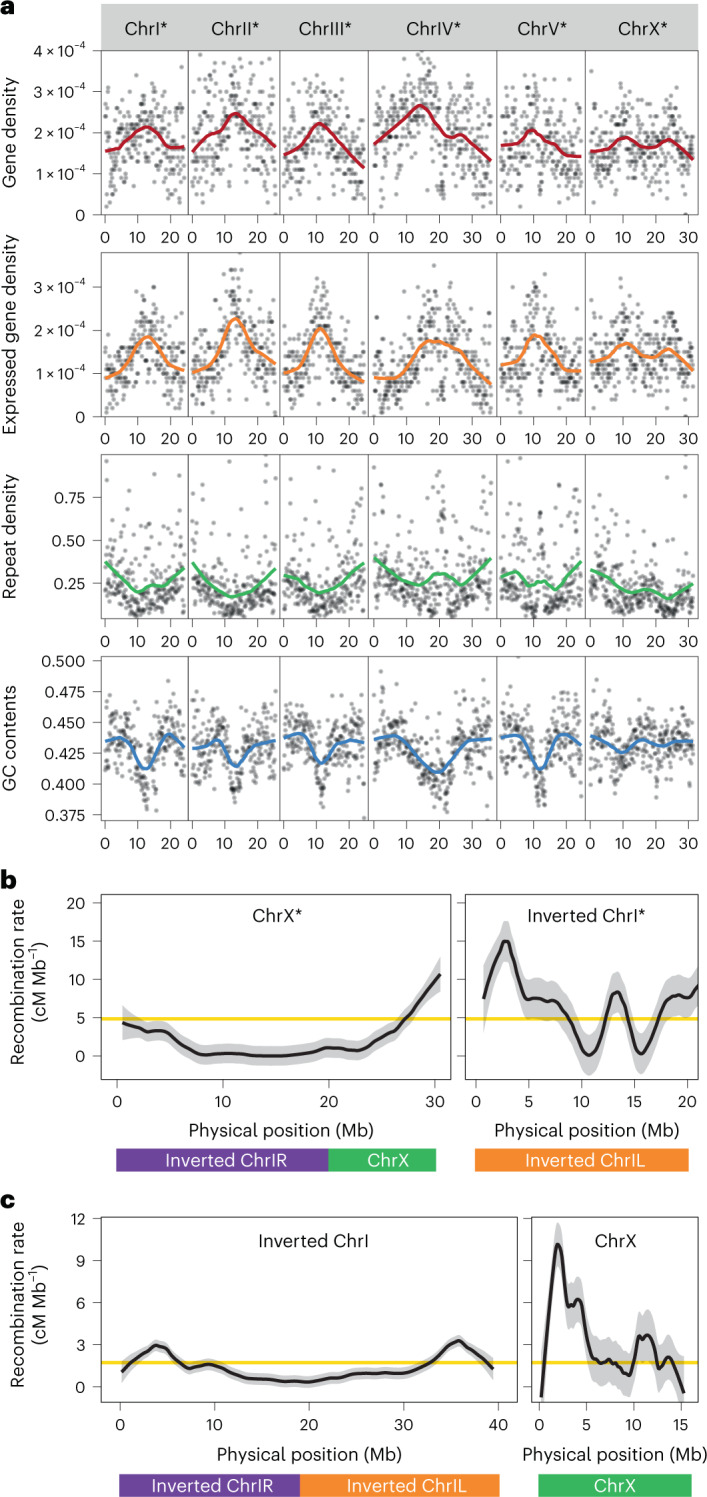
Repatterning of recombination rate in association with chromosome fusions. **a**, Genome statistics of the *P. exspectatus* genome. Plots indicate statistics of 100 kb sliding window. Coloured lines indicate LOESS fitting curve with span = 0.4. The number of genes over the sequence length was defined as gene density. Expressed gene density indicates the density of genes with non-zero median transcripts per million. The proportion of repeat-masked regions with RepeatScout pipeline was defined as repeat density. **b**, Recombination-rate pattern of the three elements ChrIL, ChrIR and ChrX in *P. exspectatus*. **c**, Recombination-rate pattern of the three elements ChrIL, ChrIR and ChrX in *P. pacificus*. ChrI or ChrI* was inverted to compare the corresponding coordinates between two species. The homologous region of the three elements is shown in the bar at the bottom. The recombination rates between neighbouring genetic markers were fitted to LOESS curve with span = 0.2 and are shown in black lines with the 95% confidence intervals shown as a grey ribbon. The average recombination rate across the genome is shown with yellow lines.

**Extended Data Fig. 6:**
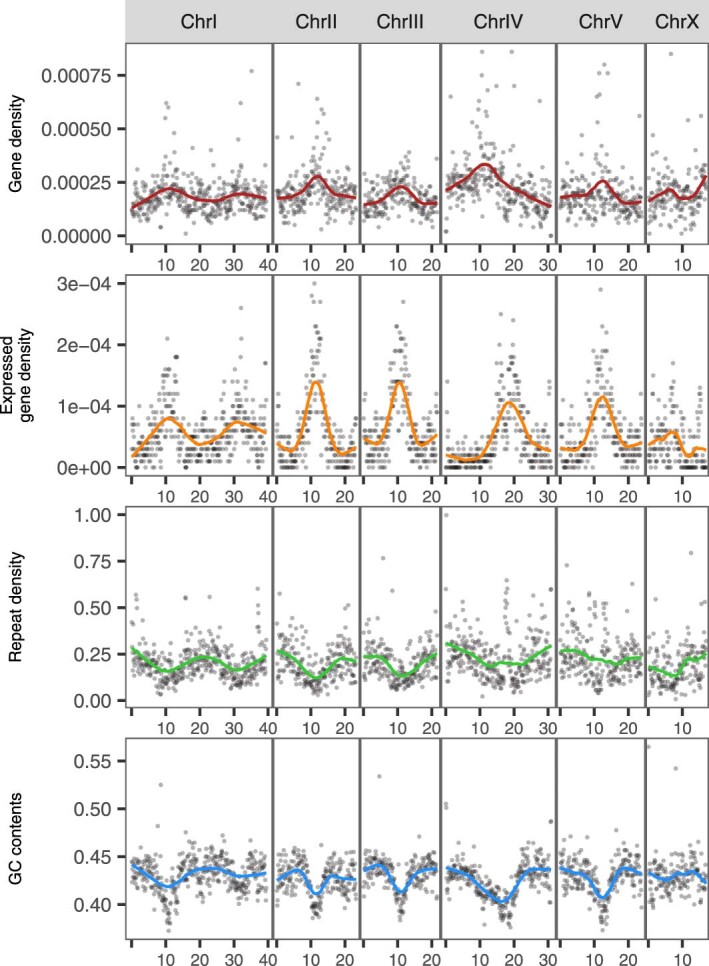
Genome statistics of the *P. pacificus* genome. Plots indicate statistics of 100 kb sliding windows. Colored lines indicate LOESS fitting curve with span = 0.4. Expressed gene density indicates the density of genes with the more than zero median TPM in pooled young-adult males (*N* = 3) and hermaphrodites (*N* = 3). Repeat density indicates the density of repeat-masked regions with RepeatScout pipeline.

To obtain further insight into the consequences of chromosome fusions, we examined the predicted gene density, expressed gene density, repeat density and guanine–cytosine (GC) content across all six chromosomes of *P. exspectatus* (Fig. 3a) and *P.* *pacificus* (Extended Data Fig. 6). As for other nematodes, *Pristionchus* species have holocentric chromosomes^44^, and consistently, the chromosomal patterns of these statistics were generally consistent with those found in other nematodes. In general, chromosome centres have a higher gene density, a low repeat density and an AT bias. However, the fused elements ChrIR and ChrX of *P. exspectatus* (ChrX* in Fig. 3a) and ChrIR and ChrIL of *P. pacificus* (ChrI in Extended Data Fig. 6) still keep patterns as if they were different chromosomes. This was consistent with previous reports in *P. pacificus*^36,45^. These patterns are also consistent with the notion that both fusion events occurred in the range of several million generations.

## Recombination-rate repatterning by chromosome fusions

**Extended Data Fig. 7:**
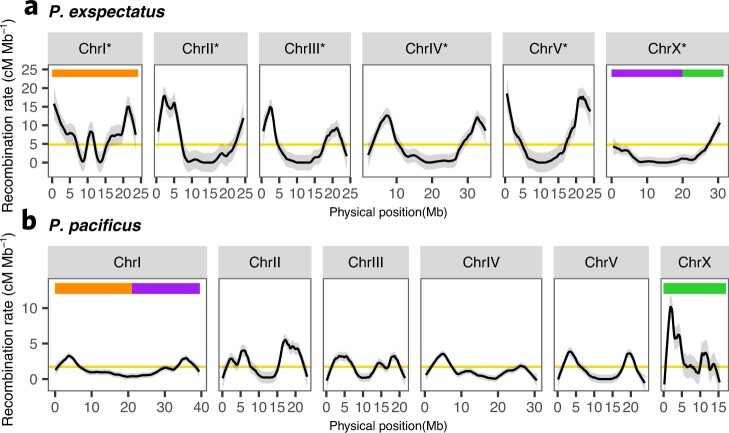
Genomic pattern of recombination rate of *P. exspectatus* (a) and *P. pacificus* (b). The three genomic regions ChrIL, ChrIR and ChrX are shown in orange, purple and green bars at the top, respectively. The recombination rate between neighboring genetic markers were fitted to LOESS curves with span = 0.2 and are shown in black lines. The 95% confidential intervals of the fitting curve are shown as grey ribbons. The average recombination rate across the genome is shown as yellow lines.

In nematode genomes, chromosome centres have reduced recombination rates^36,46^. We hypothesized that the chromosome fusions in *P. exspectatus* and *P. pacificus* have repatterned the chromosome-wide recombination rate in contrast to conserved patterns of gene or repeat density. To test this hypothesis, we experimentally investigated the recombination rate in *P. exspectatus*. As only one isolate of this species is available, we generated ethyl methanesulfonate mutants of *P. exspectatus* and crossed two mutagenized lines with each other to perform intra-strain linkage mapping (*N* = 103). For comparison, we reanalysed the recombination rate of *P. pacificus* using the published recombinant inbred line (RIL) data of two strains of *P. pacificus*^47^ (*N* = 96). The Marey map (plot of physical distance (Mb) versus genetic distance (centimorgans (cM))) and the estimate of recombination rate (cM Mb^–1^) of all chromosomes are shown in Supplementary Fig. 4 and Extended Data Fig. 7, respectively. These results indicate low recombination around the centres of chromosomes except ChrI* in *P. exspectatus* and ChrX in *P. pacificus* (Fig. 3b,c). Thus, the two fused chromosomes have the largest low-recombination regions, indicating repatterning of recombination rates after chromosome fusion. It is important to note that these extended low-recombination regions in the fused chromosomes contain more than 14.8% of predicted genes or 17.1% of expressed genes across the entire genome. We performed bootstrap sampling in which the genomic regions with the same genetic distance were randomly sampled out of the low-recombination regions to test the percentage of genes in the null distribution. These percentages were significantly higher except for predicted genes in *P. pacificus* (bootstrap test, *N* = 10,000; predicted and expressed genes in *P. exspectatus* and expressed genes in *P. pacificus*, one-tailed *P* < 0.0001; predicted gene in *P. pacificus*, one-tailed *P* > 0.05; Supplementary Fig. 5). Thus, chromosome fusions restrict recombination between large numbers of genes that can facilitate the coevolution of genes in the region as previously proposed for inversions^48^. Such coevolution of genes is thought to be an important source for hybrid incompatibility^49^.

## *P. exspectatus* has a young Y chromosome

**Extended Data Fig. 8:**
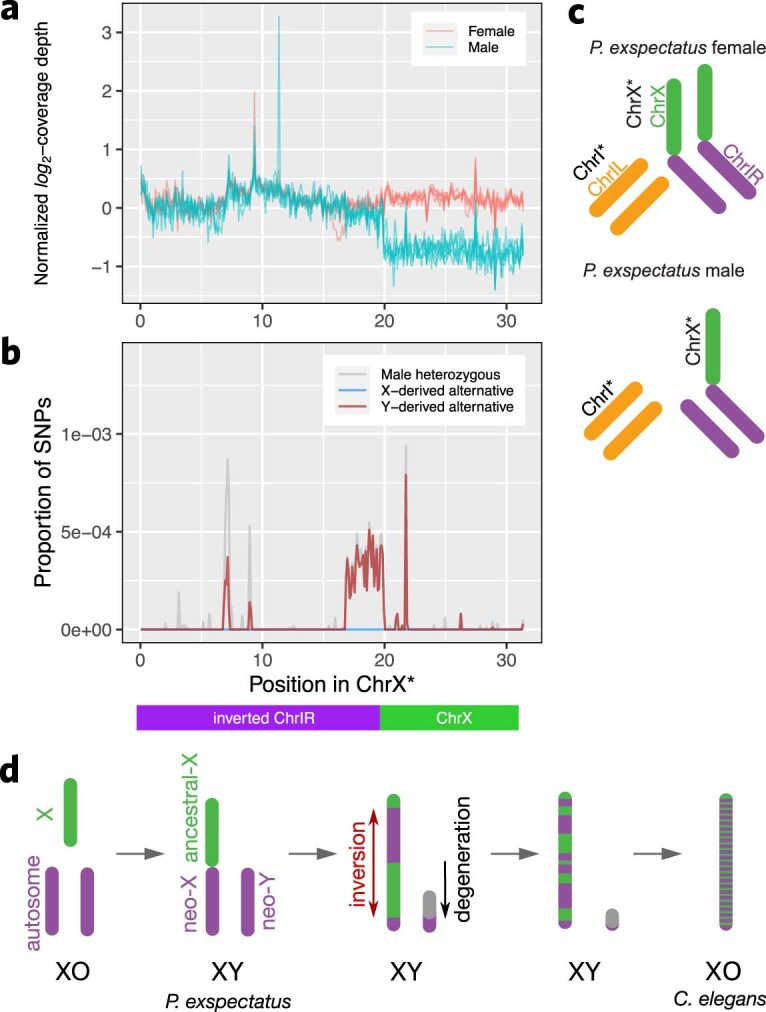
Young XY system in *P. exspectatus*. **a**, Coverage analysis of the X chromosome between sexes of *P. exspectatus*. Coverage depth of each 100 kb sliding window was normalized by average coverage and log_2_-transformed. Each lines indicates the values of sliding windows of a single individual. **b**, Increasing Y-derived alternative SNPs around the fusion breakpoint. Definitions of male heterozygous SNPs, X-derived alternative SNPs and Y-derived alternative SNPs are found in Materials and Methods and Supplementary Fig. 22. The proportion of SNPs in 100 kb sliding windows are shown. **c**, Diagram of the XY system of *P. exspectatus*. **d**, Evolutionary model of sex chromosome evolution in nematodes, consistent with the ref. ^87^. First, chromosome fusions of an X chromosome and an autosome in a XO system results in an XY system containing neo-X and neo-Y chromosome regions. Second, inversions occur between the neo-X and ancestral-X chromosomes. Restricted recombination between neo-X and neo-Y chromosomes results in the gradual degeneration of the neo-Y chromosome. Third, additional inversions accumulate in the X chromosome and the Y chromosome becomes smaller by further degeneration. Finally, the X chromosome has mixed fractions of ancestral-X and neo-X chromosomes (see Extended Data Fig. 4c), and the Y chromosome is completely lost. This final step results in the reoccurrence of the XO system. Note that the chromosome fusions of *P. exspectatus* and *C. elegans* happened independently and their sex chromosomes represent different stages in nematode sex chromosome evolution.

Further analysis revealed that the fused chromosome of *P. exspectatus* is similar to the arrangement in the ancestor of *Caenorhabditis*^36,50^. Specifically, protein-based comparative synteny analysis between *P. pacificus* and *C. elegans* (Extended Data Fig. 4b) or between *P. exspectatus* and *C. elegans* (Extended Data Fig. 4c) indicates apparently identical chromosomes between *C. elegans* and *P. exspectatus*, although the two elements, *P. pacificus* ChrIR and ChrX, are highly rearranged in *C. elegans*. Most important, the recent chromosome fusions between the autosome and the X chromosome in *P. exspectatus* formed a neo-sex chromosome. When an X chromosome fuses to an autosome in XO sex-chromosome systems, the fused sister chromosome of the autosome becomes a neo-X chromosome while another sister chromosome becomes a neo-Y chromosome, which is possessed only by males (see purple region in Extended Data Fig. 8b). Indeed, coverage analyses of genome sequences of *P. exspectatus* females and males (*N* = 5 each) show that the element shared between the X chromosomes of *P. pacificus* and *P. exspectatus* (*P. pacificus* ChrX) has a two-fold female bias reflecting hemizygosity in males (Extended Data Fig. 8a) that is consistent with the assumption of a conserved X chromosomes in nematodes^50^. By contrast, the region homologous to *P. pacificus* ChrIR has the same coverage depth in males and females of *P. exspectatus*. These results indicate that *P. exspectatus* contains a non-degenerated Y chromosome (Extended Data Fig. 8c). Note that we also detected putative Y-specific mutations around the fusion breakpoint of ChrIR (‘Y-derived alternative SNPs’ in Extended Data Fig. 8b), which are similar to neo-sex chromosomes in other organisms (for example, stickleback^51^). Finally, the karyotype analysis indicated that *P. exspectatus* sperm have a stable number of chromosomes, in contrast to *P. pacificus* and *P. occultus* (Extended Data Fig. 5b), confirming the existence of an XY system in *P. exspectatus*.

## Major QTL for hybrid sterility in the fused chromosome

**Fig. 4:**
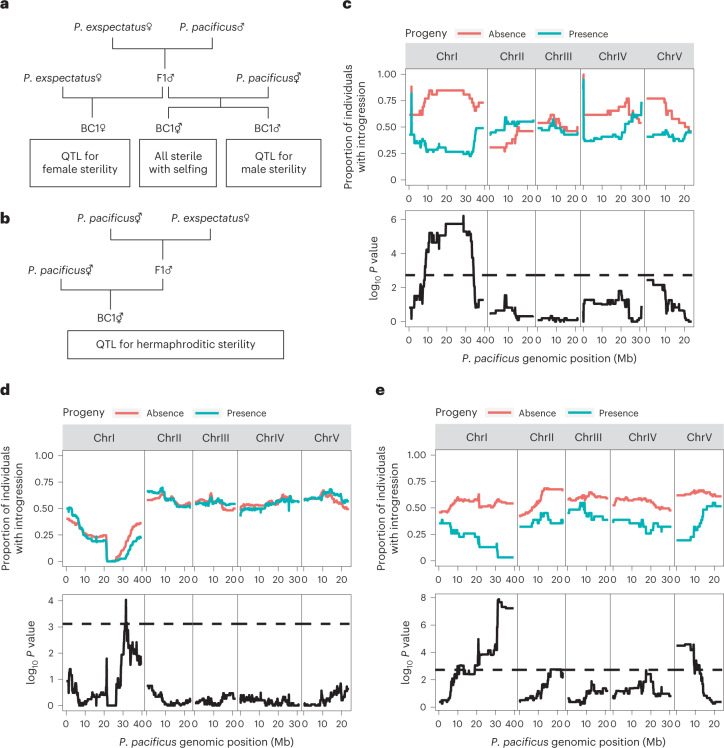
QTL study of hybrid sterility of different sexes identify major QTL at the chromosome fusion. **a**,**b**, Crossing scheme for the QTL analysis for female and male sterility (**a**) and hermaphrodite sterility (**b**). **c**–**e**, Results of QTL analysis for female (**c**), male (**d**) and hermaphrodite (**e**) sterility. Upper rows indicate proportion of individuals with introgression of each 100 kb sliding window for individuals with at least one progeny (green) or no progeny (red). In the lower rows, the log_10_ *P* value of Fisher’s exact test of association of fertility and introgression is shown as solid line. Significant threshold of *P* value of the Fisher’s exact test calculated by permutation test (*N* = 1,000, significant level = 1%) is shown as dashed line.

To test the effects of both chromosome fusions on reproductive isolation, we conducted QTL analysis for hybrid sterility of the three different sexes (hermaphrodite, female and male). We used backcrosses of fertile F1 males as indicated in the crossing scheme summarized in Fig. 4a,b (for details, see Methods and Supplementary Table 3). As females and hermaphrodites are morphologically indistinguishable, backcrossed individuals with female morphology were treated as BC1 females or BC1 hermaphrodites in the backcross to the dioecious (*P. exspectatus*) or the androdiecious (*P. pacificus*) species, respectively. The fertility of BC1 females and males was tested with an additional backcross against the parental species, whereas the fertility of the BC1 hermaphrodites was tested by selfing. However, we did not observe any progeny in BC1 hermaphrodites in one reciprocal cross (*N* = 25; Fig. 4a), while at least 22% of BC1 hermaphrodites produced progeny in another reciprocal cross (*N* = 136; Fig. 4b). Therefore, we conducted QTL analysis for hermaphrodite sterility using F1 males from crosses between *P. exspectatus* males and *P. pacificus* hermaphrodites. Note that ChrX regions cannot be analysed in the QTL analysis because the ChrX region does not have any genetic variation originating from F1 hybrid males.

After fertility testing, we lysed each BC1 animal for single-worm whole-genome sequencing. The sequence reads of BC1 animals were mapped to the *P. pacificus* reference genome for genotyping using a 100 kb sliding-window approach across the entire genome (Supplementary Fig. 6). The association between the presence/absence of progeny and presence/absence of introgression was tested in each window to detect significant QTL (BC1 female, *N* = 75; BC1 male, *N* = 291; BC1 hermaphrodite, *N* = 136; Fisher’s exact test with significance calculated in permutation test) (Fig. 4c–e). Note that the presence/absence of progeny is affected not only by hybrid sterility but also by early hybrid lethality of the progeny. However, it is unlikely that hybrid lethality alone explains the phenotype because the introgression did not affect lethality of the BC1 generation. When searching for introgression of *P. exspectatus* alleles in the backcross to *P. pacificus*, or vice versa, we found major QTL for hybrid sterility in all three sexes in the fused chromosome region. Specifically, we found a single QTL for female sterility located on ChrI as a broad peak (8.45–34.00 Mb; Fig. 4c). A single QTL for male sterility is also located on ChrIR (ChrI 31.6 Mb; Fig. 4d). Two QTLs for hermaphroditic fertility were found on ChrIR and ChrV. Note, however, that the QTL on ChrI showed much higher significance than the one on ChrV (Fig. 4e). We also conducted QTL mapping using R/qtl^52^ (version 1.48.1) and found consistent results and QTL peaks (Supplementary Fig. 7). Again, the QTL for hermaphrodite sterility on ChrI showed higher percentage of variance (PVE) than the QTL on ChrV (PVE of the ChrI peak, 20.1%; PVE of the ChrV peak, 10.0%). Note that we use different experimental schemes for QTL analyses (Methods) to avoid contamination of self-progeny of parental hermaphrodites and to sample sufficient replicates. These differences make it difficult to compare the number and length of QTL peaks between sexes. However, although the experiment was conducted differently, we reached the same conclusion that a major QTL for hybrid sterility is located in the region of the chromosome fusions, ChrIR.

## Aneuploidy inducing hybrid female sterility

**Fig. 5:**
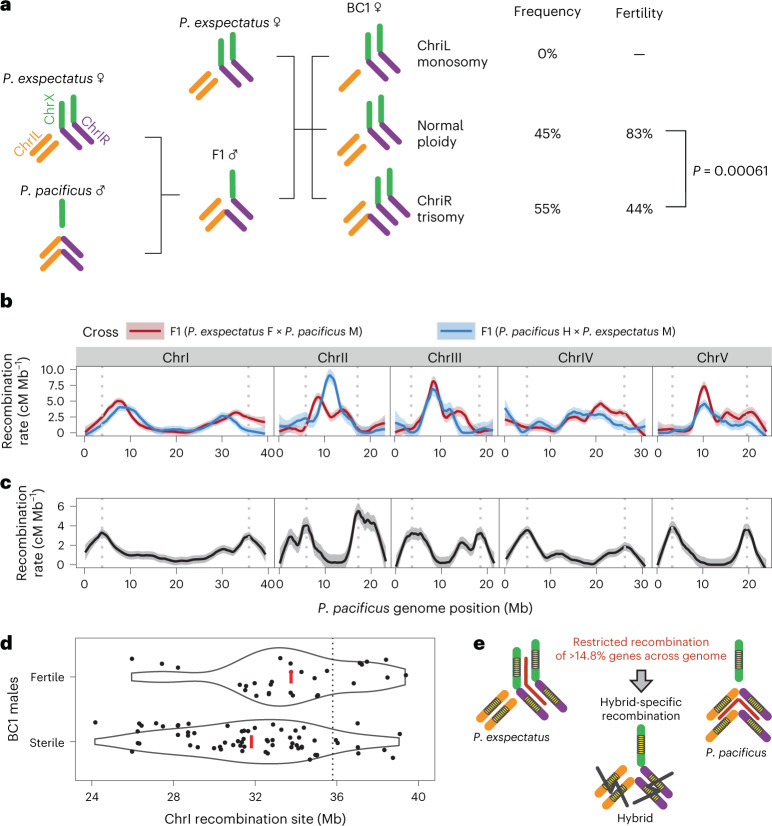
Multiple effects of chromosome fusions on the evolution of hybrid sterility. **a**, Chromosomal fusions induced trisomy that is associated with hybrid female sterility. The *P* value of Fisher’s exact test is shown (see also Supplementary Table 5). **b**,**c**, Genomic pattern of recombination rate of the interspecies (**b**) and intraspecies (**c**) F1 individuals. BC1 male genotype data were used for the recombination rates of F1 of *P. exspectatus* females versus *P. pacificus* males (red in **b**), while BC1 hermaphrodite genotype data were used for the recombination rates of F1 of *P. pacificus* hermaphrodite versus *P. exspectatus* males (blue in **b**). RILs data of *P. pacificus* strains PS312 versus RSB001 were used for the intraspecies recombination rate (black in **c**). The recombination rates between neighbouring genetic markers were fitted to LOESS curve with span = 0.2 and are shown in solid lines with the 95% confidence intervals shown as light-coloured ribbons. The grey vertical dotted lines show the peak of recombination rate at both chromosome ends in the intraspecies hybrid meiosis. **d**, Association between shifted recombination rate and BC1 male sterility. Violin plot for recombination sites between BC1 males with presence/absence progeny. The plots indicate the distribution of recombination sites of BC1 males having recombination in the region. The red line shows the mean position of recombination sites. The dotted line indicates the peak of recombination rate of *P. pacificus*. **e**, Hybrid-specific recombination-breaking gene combination of low-recombination regions surrounding chromosome fusions.

**Extended Data Fig. 9:**
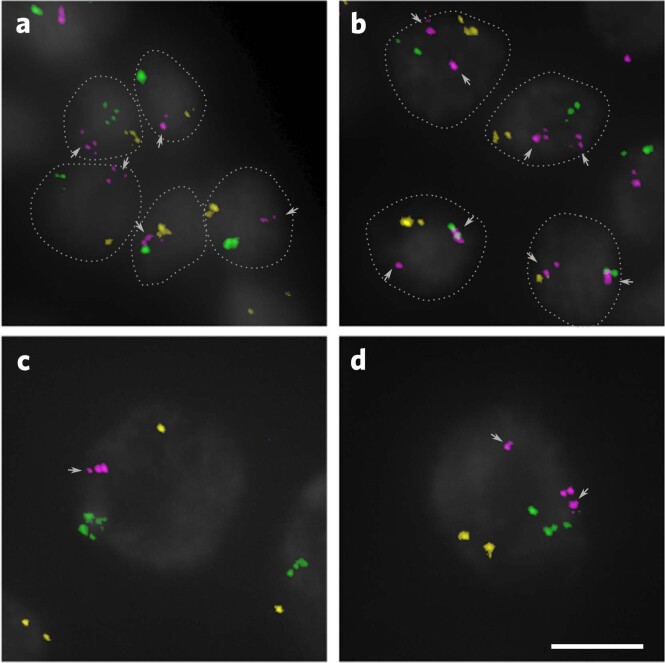
FISH analysis of meiotic cells of BC1 females with the specific *21*-mer of the three elements, ChrIL (yellow), ChrIR (magenta), and ChrX (green). Earlier (**a**, **b**) and later (**c**, **d**) stage of Prophase I cells of different individuals are shown. DAPI staining is shown in grey. **a**, **b**, single nuclei are circled by dotted lines. The arrow indicates the cluster of signals of ChrIR. The additional FISH signals of ChrIR were distantly located in 3 out of 10 individuals (e. g. **b**, **d**), whereas only one cluster was found in the others (e. g. **a**, **c**). Such a variation did not happen with ChrIL and ChrX. These pictures were obtained in the Z-stack projection. The scale bar is 5 µm.

Chromosome fusions were proposed to cause reproductive isolation via unbalanced segregation in F1 meiosis that produces F1 hybrid sterility or aneuploidy in the next generation^5,6^. In hybrids between *P. exspectatus* and *P. pacificus*, such unbalanced segregation may result in trisomy or monosomy of ChrIR in BC1 and explain their sterility (Fig. 5a and Supplementary Fig. 8). To test this hypothesis, we analysed the normalized coverage of sequence reads of the seven chromosomal regions (ChrIL, ChrIR and the other chromosomes) to estimate the ploidy in BC1 hybrids (Supplementary Fig. 9). Indeed, we were able to detect increased coverage in the expected elements in BC1 sexes, suggesting frequent trisomy, but no monosomy (Fig. 5a and Supplementary Fig. 9). We identified a significantly high percentage of trisomy in almost all of the expected chromosomes (Supplementary Table 4). FISH analysis of Prophase I cells of the BC1 females also confirmed the additional signals of ChrIR in three out of ten individuals (Extended Data Fig. 9). Next, we tested for a potential association of trisomy with sterility and found that BC1 female sterility can be at least partially explained by trisomy (Fisher’s exact test, two-tailed *P* = 6.1 × 10^−4^; Fig. 5a and Supplementary Table 5). By contrast, the other sterilities were not explained by trisomy (BC1 male, two-tailed *P* = 0.56; BC1 hermaphrodite, two-tailed *P* value of ChrIL and ChrIR trisomy = 0.38 and 0.52, respectively; Supplementary Table 5). These results suggest that the chromosome fusions result in hybrid aneuploidy that contributes to reproductive isolation, but the accumulation of QTL associated with the chromosome fusions cannot fully explain this pattern.

## Hybrid-specific recombination induced hybrid male sterility

**Extended Data Fig. 10:**
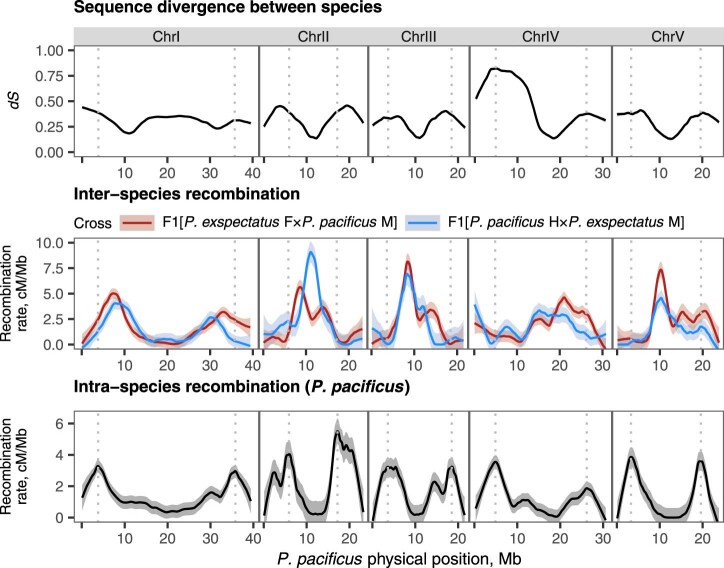
Sequence divergence calculated by normalized synonymous-mutation rate negatively correlated with recombination rate in hybrids. Because alignments often fail in non-coding regions especially at the chromosome periphery, synonymous-mutation rate of the coding region was used to calculate sequence divergence. Genomic pattern of recombination rate of the inter and intraspecies hybrids is shown for comparison.

The independent chromosome fusions in *P. exspectatus* and *P. pacificus* resulted in long low-recombination regions that contain >14.8% of all genes (Fig. 3b,c). These regions can accumulate epistatic mutations, which might lead to hybrid abnormality when hybrids have recombination within the regions. The availability of single-worm whole-genome sequencing data of hybrid animals allows testing this hypothesis by analysing the distribution of the recombination rate in F1 hybrid meiosis. Note that we removed potential trisomy for this analysis (BC1 male and hermaphrodites after the removal, *N* = 216, and *N* = 92, respectively; Fig. 5b and Supplementary Fig. 4c,d). Strikingly, the recombination pattern of hybrids was clearly different from that of both parental species (Fig. 5b,c and Supplementary Fig. 4). In general, the peak of recombination was shifted towards the centre in any chromosomes (Fig. 5b). In some chromosomes, the highest recombination rates were seen in the chromosome centre (Fig. 5b). Such a pattern has never been observed in any nematode before, although the negative correlation between sequence divergence and recombination between species (Extended Data Fig. 10) suggests that the pattern can be explained by the loss of sequence homology in the gene-poor chromosome peripheries. In addition, some of these gene-poor regions have inversions (Supplementary Fig. 3) that partially explain hybrid-specific recombination. This hybrid-specific recombination might break the linkage of genes in the low-recombination region in hybrids and cause hybrid abnormalities (Fig. 5e). Indeed, when we compared recombination sites between BC1 males and tested their association with sterility we found that males that did not give progeny had more recombination sites in the centre of ChrIR (Fig. 5d). Using a generalized linear model, we confirmed a significant reduction of fertile animals with shifted recombination sites towards the chromosome centre (*N* = 93, coefficient of recombination site = 0.1777; likelihood ratio test between an intercept-only model and an alternative test model with recombination sites included as explanatory variable, likelihood ratio statistics = 6.65, *P* = 9.9 × 10^−3^; Supplementary Fig. 10). We conclude that the physical shift of recombination sites is associated at least with male sterility. Taken together, the analysis of hybrids between *P. exspectatus* and *P. pacificus* provides empirical support for a role of chromosome fusions in hybrid sterility.

## Discussion

This study revealed differences in genome structure between closely related nematodes, indicating (1) chromosome fusions involving the same ancestral chromosome (Fig. 2), (2) genetic evidence for the effect of chromosome fusions on reproductive isolation (Fig. 4) and (3) an evolutionary mechanism of reproductive isolation by chromosome fusions (Fig. 5). Thus, we provide new insight into the role of chromosome fusions in speciation. Early theoretical hypotheses proposed that chromosome fusions directly contribute to reproductive isolation, but they were not commonly confirmed in experimental studies^5–7^. Instead, more-recent hypotheses proposed that the reduction in recombination by chromosomal rearrangements results in coevolving genes reducing gene flow^7,9,22,48^, which was empirically confirmed for inversions^12,14–20^.

**Fig. 6:**
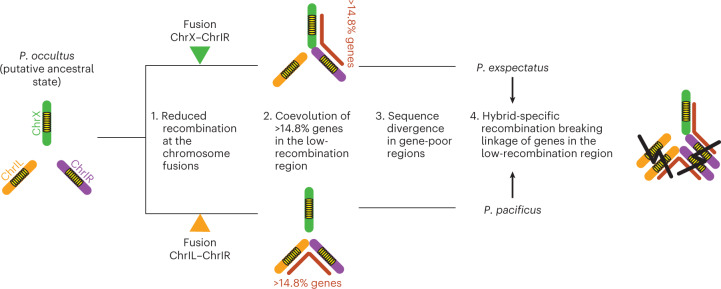
Evolutionary model of the effect of chromosome fusions on reproductive isolation via recombination repatterning. Yellow ovals indicate gene-rich regions at the centre of chromosomes. Red lines indicate the low-recombination regions resulting from chromosome fusions.

Our study suggests multiple independent evolutionary effects of chromosome fusions in speciation of closely related nematodes (Fig. 5). First, we observed abnormal chromosome segregation in F1 hybrid meiosis that led to aneuploidy (Fig. 5a), which is in agreement with classical hypotheses and partially explains hybrid female sterility. Second, we found that recombination reduction of the fusion breakpoints formed large low-recombination regions in the pure species. These regions experienced recombination in hybrids, and hybrid-specific recombination explains hybrid male sterility (Fig. 5d). We propose an evolutionary model described in Fig. 6. The ancestor of the two current species presumably had seven chromosomes, similar to the pattern seen in *P. occultus* with ChrIL, ChrIR and ChrX as separate chromosomes. Chromosome fusions involving ChrIR occurred independently in the lineage leading to *P. pacificus* and *P. exspectatus*. These fusions were partially underdominant because of meiotic abnormalities, but the underdominance might not have been large enough to prevent the fixation of these fusions. Alternatively, these fusions might have captured beneficial allele combinations to cancel the underdominance. The chromosome fusions altered the recombination rate of the fused chromosomes, resulting in long low-recombination regions in which genes can coevolve. In hybrids, sequence divergence and/or inversions in gene-poor regions probably shifted the recombination towards the low-recombination regions that break allele combination in these regions, resulting in hybrid abnormality. Note that we do not know whether the fusion in *P. exspectatus* is fixed in the population. However, this study also indicated that *P. pacificus* and *P. exspectatus* are nascent species that are not completely reproductively isolated, and the available isolate of *P. exspectatus* is from a geographic region where *P. pacificus* is also found. Therefore, these fusions currently contribute to maintain reproductive isolation between species (Supplementary Note). In addition, we do not know the recombination rate of the heterokaryotype immediately after the occurrence of the chromosome fusions because heterokaryotypes observed in this study are from hybrids of species with sequence divergence. Besides additional taxon sampling, future studies using an artificial introduction of fusions at the target site in *P. pacificus* can test the effect of chromosome fusions as suggested by theory.

The fusion between an autosome and the sex chromosome in *P. exspectatus* made the ChrIR region a neo-sex chromosome. It has been proposed that the ancestor of *Caenorhabditis* species also experienced the same fusion^50^, which is consistent with our study (Extended Data Fig. 8). *Caenorhabditis* species, however, have an XO sex-chromosome system where the sex chromosome is hemizygous among distantly related *Caenorhabditis* species^42,43^. Therefore, we speculate that the same chromosome fusion event occurred early in the evolutionary history of *Caenorhabditis* and was followed by the loss of the Y chromosome through Y-chromosome degeneration (Extended Data Fig. 8d). By contrast, the Y chromosome in *P. exspectatus* is of recent origin and did not yet degrade. Thus, *P. exspectatus* allows studying the initial stages of such sex-chromosome evolution. Interestingly, X and Y chromosomes are diverged only at the end of the neo-sex-chromosome region, but Y-specific mutations were found also in other small regions (see ~22 Mb of Extended Data Fig. 8b). This might suggest that some genomic regions out of ChrIR were duplicated to the Y chromosome.

The important role of sex chromosomes in reproductive isolation has been observed across taxa^8^ (‘large-X effect’), and it is proposed that the formation of neo-sex chromosomes promotes evolution of genes with a role in reproductive isolation^53^. Indeed, our study revealed a major QTL for hybrid sterility in the region of the neo-sex chromosome where X and Y are differentiated only in a limited region. While a similar accumulation of QTL for reproductive barriers in the neo-sex-chromosome region was observed in stickleback^51,54^, there might be additional mechanisms that promote hybrid incompatibility in the ChrIR region in *Pristionchus*.

We also observed intrachromosomal rearrangements that differentiate the genomes of the two species and partially explain hybrid-specific recombination. However, we could not detect any QTL peak in these regions. The divergence of the highly structured regions can be explained by relaxed selection against synteny, which is consistent with the frequent occurrence of inversions in gene-poor regions. Future studies using genome assemblies of additional strains of the two species might reveal whether the inversions are fixed within species and whether they played a role in speciation.

Chromosome fusions were previously not considered to represent a major factor of species divergence in nematodes. One of the reasons is that the well-studied genus *Caenorhabditis* has conserved karyotypes^41,43,55–57^. However, recent comparative genomic analyses indicate chromosomal diversity of nematodes outside of *Caenorhabditis*^36,50,58–62^ (see detail, Supplementary Note). For example, work in the two distantly related human parasitic nematodes, *Brugia malayi* and *Onchocerca volvulus*, indicated chromosome fusions, where two ancestral chromosomal regions still keep partitions within chromosomes as in *Pristionchus*^50,60^. Interestingly, the two parasitic nematodes have fusions between an X chromosome and the homologous regions to ChrIR or ChrIL^50^. Therefore, we hypothesize that similar speciation mechanisms might exist in several taxa, but the recombination mechanisms might be specific to organisms with holocentric chromosomes.

In summary, convergent mechanisms in particular involving the chromosomes ChrIL, ChrIR and ChrX might indicate evolutionary trends of repeated chromosome fusions. Our data, together with the recent finding of chromosome fusions between distinct taxa of nematodes^50^, make us speculate that the same phenomena occur not only in different nematode groups but also in very short evolutionary time frames. Therefore, future work in *Pristionchus* can investigate the diversity of karyotypes and its relationship with speciation in *Pristionchus*. The evolution of reproductive isolation is explained not only by simple interactions of genes, but also by complex genome instability, including chromosome instability^63^. *Pristionchus* with its large and growing number of species, the ability to perform crossing experiments that form viable hybrids and the available molecular tool kit represent a promising system to study the evolutionary causes and consequence of chromosome fusions and other genomic changes during speciation.

## Methods

### Chromosome nomenclature

The chromosome regions ChrIL and ChrIR are defined as the homologous regions to the left and right of position 21.05 Mb of chromosome 1 in *P. pacificus*, respectively. The three chromosomal elements ChrIL, ChrIR, and ChrX regions correspond to the conserved chromosome elements recently named as NigonE, NigonN and NigonX, respectively^59^ (see detail, Supplementary Note). Note that we refrained from using this terminology as it was differently applied in recent publications^50,60^.

### Crossing experiments for reproduction tests

The first crossing experiments were performed as previously reported^31^. In short, three J4 juvenile females of dioecious species and six young-adult males of *P. pacificus* were crossed on Nematode Growth Medium (NGM) plates with a 50 µl OP50 lawn (*N* = 4 in each species). The following strains were used: *P. pacificus*, PS312; *P. exspectatus*, RS5522; *P. occultus*, RS5811; *P. sikae*, RS5901; *P. arcanus*, RS5527; *P. kurosawai*, RS5914; *P. taiwanensis*, RS5797; *P. maxplancki*, RS5594. Males were removed after two days and females were removed after four days to avoid backcross. Hybrids were allowed to cross on the same plate and cultured. To prevent starvation, at least 50 worms were transferred to a new plate to continue the culture. If newborn larvae continued to be produced for one month (~6 generations), we considered the hybrids to propagate. To confirm the reproducibility of the original cross between *P. pacificus* and *P. exspectatus*, we made eight additional replicates (Supplementary Table 6). To prevent starvation, 25% of the individuals on the plate were transferred before starvation (after day 5). Thus, the number of animals counted on day ten is reduced relative to day five because 75% of the animals were not transferred. Subsequently, >95% of worms were transferred in the second or later transfers. We counted the number of individuals all 5 days for 30 days, indicating the numbers of J2/J3 and J4 juveniles, as well as adults for both, hermaphrodites and males. Note that eggs were not counted. We found continuous production of juveniles in four of the eight replicates (50%). Note that the number of animals declines over time because some hybrid progeny die without producing a large number of progeny. Importantly, however, new juveniles were constantly observed throughout the duration of the experiment, and no trend of change of sex ratio was observed (Supplementary Table 6).

For quantitative reproduction tests, one virgin female and one young-adult male were mated on the NGM plate with a 50 µl OP50 lawn with egg laying for six days. Parents were transferred to new plates every second day. Progeny were grown for three to four days on these plates. The number of males, females (or hermaphrodites) and immature progeny were counted on the basis of their morphology. Because the hermaphrodites have the same morphology as females, we do not distinguish these two sexes. When the two-arm gonad and the vulva were observed, the worm was categorized as a female or hermaphrodite. When the one-arm gonad and connection of the gonad to the spicule were observed, the worm was categorized as a male. When these reproductive traits were not observed, the worm was categorized as an immature animal. The type strain of *P. pacificus*, PS312, and an inbred line of the type strain of *P. exspectatus*, RS5522B, were used in this experiment. For hybrid crosses, old females or hermaphrodites (four days after J4 stage) were used for mating to let hermaphrodites run out of self-sperm. We also tested the number of progeny of old *P. pacificus* hermaphrodites without mating at the same time (*N* = 18). Because only one progeny was found from all hermaphrodites (0.056 progeny per replication on average), the self-progeny is negligible in the analysis. For backcrossing, we first prepared F1 animals produced by *P. exspectatus* dam and *P. pacificus* sire or F1 animals produced by *P. pacificus* dam and *P. exspectatus* sire using the experimental set-up described in the preceding and backcrossed them with animals of the pure species. We used young J4-stage females or hermaphrodites for backcrossing. We did not test the backcross with *P. pacificus* hermaphrodites that produce ~200 self-progeny because that makes the interpretation difficult. For the test of hermaphroditic reproduction of F1 animals, F1 female or hermaphrodite was placed on the NGM plate with a 50 µl OP50 lawn without males. We tested the wild-type cross of *P. exspectatus* in each backcross as control. For intercrosses of F1 animals, F1 hybrids were crossed to each other to avoid the effect of inbreeding. The sample number of each experiment is listed in Supplementary Table 1. Asymptotic Wilcoxon–Mann–Whitney test was performed using wilcox_test function of an R package, coin.

The reproductive capacity of F1 males was compared between crosses of *P. pacificus* and different dioecious species using the same experimental scheme. The F1 males were produced by crosses between females of dioecious species and *P. pacificus* male and backcrossed with parental dioecious species. We used wild isolates of six dioecious species. Only presence or absence of progeny (BC1) was analysed in these experiments (*N* = 25 each).

### De novo assembly of the *P. exspectatus* genome

Sample preparation for PacBio sequencing of *P. exspectatus* (RS5522B) was performed according to the manufacturer’s instructions (101-693-800 version 01). The strain was previously inbred for ten generations, which cannot completely exclude the possibility of remaining heterozygosity. Genomic DNA was extracted from worms taken from ~300 starved plates using QIAGEN Genomic DNA Maxi kit (#13362, QIAGEN), qualified by Nanodrop (Thermo Fisher) and quantified by Qubit (Thermo Fisher). Size fraction of the DNA was quantified using FEMTO Pulse (Agilent) before and after shearing the DNA (6.5 µg) with two strokes in a 26 G needle. The sample was sequenced in two SMRT cells of the PacBio sequencer. This yielded 21 Gb of raw sequencing data, which translates into a 120× coverage of the *P. exspectatus* genome, whose size is estimated in the following (170 Mb). Median read length was 13 kb, with an interquartile range of 5–26 kb. Raw PacBio reads were assembled using the Canu assembler^64^ (version 1.4), which resulted in a preliminary assembly with 283 contigs (230 Mb, N50 = 3 Mb). Repeat sequences were identified using a pipeline with RepeatScout^65^ (version 1.0.5) with a seed length of 14 and the minimum number of repeats of 5 and soft-masked by RepeatMasker (version 4.1.1) (http://www.repeatmasker.org/). The masked assembly was processed by HaploMerger2^40^ to build the haploid genome with 104 contigs (170 Mb, N50 = 4.1 Mb). The resulting assembly was scaffolded using Hi-C scaffolding. This resulted in the final assembly with 24 scaffolds (170 Mb, N50 = 26 Mb).

The Hi-C library was prepared from around 1,500 fresh worms of *P. exspectatus* (RS5522B) using an Arima-HiC kit (#A510008, Arima Genomics) and a Collibri ES DNA library prep kit (#A38605024, Thermo Fisher) according to the manufacturers’ protocols. The library was sequenced using a MiSeq instrument with the MiSeq reagent kit v3 (#MS-102-3001, 101 cycles × 2). Out of the 7.6 million pairs of short reads (1.44 Gb) obtained from MiSeq, 7.4 million pairs (97.7%) were successfully mapped to the draft assembly with BWA-MEM^66^ (version 0.7.17). The mapped reads were processed and filtered with the Arima-HiC Mapping Pipeline (version 02) (https://github.com/ArimaGenomics/mapping_pipeline). Finally, 3.6 million pairs of reads (664 Mb), which corresponds to 3.9× coverage of the genome, were used in the analysis. According to Hi-C read information, the assembly was further scaffolded using Juicer^67^ (version 1.6) with default options and the 3D-DNA pipeline^68^ (version 180114) with option -e. The chromosome-level scaffolds were extracted by manual curation using Juicebox version 1.11.08^69^.

We validated the resulting assembly using coverage of Illumina sequence reads of five females and five males of *P. exspectatus*. The sequencing was performed as previously described with some modifications^70^. Young-adult worms were immersed in 20 or 30 µl of single-worm lysis solution (10 mM Tris-HCl, 50 mM KCl, 2.5 mM MgCl_2_, 0.45% NP-40, 0.45% Tween, 0.2 mg ml^–1^ Proteinase K, pH = 8.3) followed by treatment at −80 °C for >30 min and at 65 °C for 2 h. Only one-fourth of the lysate of a single worm was used for library preparation. Sample DNA was purified, tagmented by Tn5 transposase (#20034197, Illumina) and amplified by 14-cycle polymerase chain reaction with barcodes. After size selection of the polymerase chain reaction product, the concentration and size of the DNA were analysed by Qubit (Thermo Fisher) and Bioanalyzer (Agilent), respectively. The same molarity of DNA of multiple samples was pooled for sequencing in a single lane of Illumina Hiseq 3000 (Illumina). Sequence reads were mapped to the assembly with BWA-MEM. If the alignment score was lower than 30, the read was removed. Coverage depth was calculated with Samtools^71^ (version 1.1.0) and analysed in custom perl and R scripts. We found female-specific reduction of coverage in the middle of the X chromosomes (Supplementary Fig. 11a). Because this region may show strong divergence between X and Y chromosomes, Haplomerger might have over-merged the sequences of X and Y chromosomes.

To detect the contamination of Y-chromosome sequence in the X-chromosome assembly, we screened for ‘X-derived alternative’ and ‘Y-derived alternative’ single-nucleotide polymorphisms (SNPs) determined as follows (Supplementary Fig. 11c). When all males had at least one read of the reference and the same alternative variant in the locus, the SNPs were defined as male heterozygous SNPs. When all females had at least one read of an alternative variant but no reads of reference variant in a male heterozygous SNP, the SNP was determined as an X-derived alternative SNP. By contrast, when all females had at least one read of reference variant, but no reads of alternative variant in a male heterozygous SNP, the SNP was determined as a Y-derived alternative SNP. The X-derived alternative SNPs presumably appear only when the reference has the Y-chromosome allele and were detected in the two regions around the fusion breakpoint (Supplementary Fig. 11b). Therefore, sequences in the large region of the X chromosome (5,000,001–20,548,412 bp in the final assembly) were replaced with that of the original assembly before the Haplomerger step, which already had a long contig for the X chromosome. The resulting assembly was validated with the individual genome sequence again. The female-specific coverage reduction and X-derived alternative SNPs were not found in the assembly after the manual fixation (Extended Data Fig. 8b). In the SNP analysis described, SNPs were called using samtools mpileup^72^ with -g option and bcftools^71^ (1.8–12) with -m option. Only reads with the base quality ≥ 13 were counted.

### Gene annotation

Evidence-based gene annotation for *P. exspectatus* was generated by the Perl Package for Customized Annotation Computing annotation pipeline^73^ (version 1.0). In summary, a transcriptome assembly of *P. exspectatus*^38^ and the community-curated *P. pacificus* gene annotation^74^ (version El Paco gene annotation 3) were aligned against the *P. exspectatus* de novo assembly with the help of the exonerate alignment tool^75^ (version 2.2.0), and the longest open reading frames per 100 bp windows were chosen as representative gene models (see ref. ^73^ for further details). Assessment of annotation quality was carried out using the benchmarking of universal single-copy orthologues approach^76^ (version 3.0.1) by comparison with the nematode odb9 dataset (*N* = 982 orthologues).

### Comparative synteny analysis

For nucleotide-based comparative synteny analysis, we ran lastz^77^ (version 1.04.00) with the notransition and nogapped options and step = 20 to search for homologous sequences of two genomic sequences, the *P. pacificus* reference genome versus the *P. exspectatus* de novo assembly. For *P. pacificus*, the ‘El_Paco’ reference genome was used^36^. Only homology at unique sites in the *P. exspectatus* genome was selected. Pairs of 100 kb non-overlapping sliding windows of the two genome sequences having at least 10 kb sequence homology are visualized in the circos plot (Fig. 2a). To visualize any small homology in the dot-plot analysis, we calculated the *P. pacificus* genome coordinate homologous to any nucleotides of *P. exspectatus* as distance from the start site of homology divided by the length of homology. The plot is downsized by 1/100 for visualization. Inversions larger than 100 kb were detected manually from dot-plot analysis, and the position was identified in the 10 kb scale.

For the protein-based comparative synteny analysis, one-to-one orthologues between species were identified as best reciprocal hits with the help of the get_BRH.pl script from the Perl Package for Customized Annotation Computing package^73^. For *C. elegans*, the dataset of WormBase ParaSite release 14 (https://parasite.wormbase.org) was used.

### Karyotyping

Males were dissected in sperm salt solution (50 mM PIPES, 25 mM KCl, 1 mM MgSO_4_, 45 mM NaCl, 2 mM CaCl_2_, pH = 7) with 200 µM Hoechst 33342 (H1399, Thermo Fisher) on Superfrost Plus slide glass (15438060, Thermo Fisher) and mounted with coverslips. For each species, at least nine male gonads with diakinesis in Prophase I and gamete cells were observed for counting chromosomes using a fluorescent microscope (Imager.Z1, Zeiss). For diakinesis, chromosomes were counted in Z-stacks. For gamete cells, only cells with chromosomes aligned in the plane of focus were used.

### FISH probe

Distribution of 21-mers was analysed in each chromosome of *P. pacificus* and *P. exspectatus* using Jellyfish 2^78^ (version 2.3.0). ChrIL (ChrI ≤ 21,050,000 bp) and ChrIR (ChrI > 21,050,000 bp) of *P. pacificus* were treated as different chromosomes. The 21-mers that appear more than 50 times in one *P. pacificus* chromosome and in its homologous chromosomes of *P. exspectatus* but less than 50 times in any other chromosomes were selected as candidate markers for a given chromosome. The selected 21-mers were assembled using Geneious Prime (version 2021.0.3; Biomatters) to detect overlap of 21-mers. Some 20–24 bp oligo DNA samples were designed in the assembly with no overlap of more than 2 bp. Multiple probes were synthesized with the modification of DY415, Fluorescein and Cy3 by Eurofins (Luxemburg) with probe information being described in Supplementary Table 7.

### FISH analysis

Young-adult males (ten per slide) were dissected in 10 µl sperm salt solution on 0.01% poly-L-lysine coated glass slides. Gonads were incubated for 2 min with 0.06% Triton/sperm salt solution. We added the same amount of glyoxal fixative mix (8% glyoxal solution (#128465, Sigma Aldrich), 20% ethanol, 0.75% acetic acid, 71% H_2_O, pH = 4–5) to the solution and incubated for 4 min. Gonads were fixed on the glass slides by freezing in liquid nitrogen, immersed in methanol and stored at −20 °C. Gonads were twice washed with 2× SSCT (saline sodium citrate (SSC) with 0.1% Tween-20) for 5 min, denatured with 50% formamide/1× SSCT for 6 h, mounted with 10 µl FISH probe mix (1 µM probe oligo, 1.275 mg Dextran, 1.7 µl 20× SSC, 5.8 µl Formamid, 1.23 µl H_2_O), denatured again at 93 °C for 2 min and hybridized at 37 °C overnight. After hybridization, slides were washed with 2× SSCT for 5 min three times. Gonads were mounted with 1/100 4,6-diamidino-2-phenylindole/Vectashield and observed on the fluorescent microscope (Imager.Z1, Zeiss).

For the FISH analysis of Prophase I cells of BC1 female, BC1 female animals were prepared as described for the QTL analysis that follows. Adult BC1 females (eight per slide) were dissected in 10 µl sperm salt solution on Superfrost Plus Gold adhesion microscope slides (#K5800AMNZ, Epredia). Gonads were fixed on glass slides by freezing in liquid nitrogen, immersed in methanol and stored at −20 °C. The hybridization steps were the same as described for male gonads.

### Genome statistics

To count the number of expressed genes, gene expression was analysed using the reported RNA-seq reads of pooled young-adult females (*N* = 3) and males (*N* = 3) under laboratory condition^38^. Transcripts per million (TPM) of predicted genes of each replication were obtained by salmon^79^ (version 1.1.0) using transcript sequences of predicted genes as reference. Genes with the median TPM in six replications >0, which include genes with TPM in either all male replications or all female replications >0, were defined as expressed genes. Identification of repeat sequences was performed with the complete assembly with the RepeatScout pipeline as described previously. The proportion of masked regions was defined as ‘Repeat density’. The predicted gene density, expressed gene density and repeat density, GC contents were analysed using custom perl script and R script. For *P. pacificus*, the newest genome assembly (El_paco) and annotation (El_paco_genome_v3) were used^36,74^.

### Sample collection for QTL analysis

We conducted QTL analyses for hybrid sterility using different crossing schemes for the different sexes of BC1 to sample enough BC1 animals and to avoid contamination of self-progeny of parental hermaphrodites. The crossing scheme and number of crosses are summarized in Supplementary Table 3. For QTL analysis of female sterility, we used the BC1 females from the cross between females of *P. exspectatus* (RS5522B) and F1 males that are progeny of P0 females of *P. exspectatus* (RS5522B) and P0 males of *P. pacificus* (P312). Each virgin J4 BC1 female was crossed with a young-adult male of *P. exspectatus* to test fertility of the female for six days, and the number of hatched progeny was counted. After this, the BC1 female was lysed for genotyping.

For QTL analysis of male sterility, we used a fluorescent transgenic line, PS312Ex[*egl-20*::RFP, *tph-1*::RFP] (RS3066), previously established^80^ to isolate cross progeny from self-progeny. This line has high transmission rate (~90%) of the extra-chromosomal array of RFP with *egl-20* and *tph-1* promoters in the PS312 background. This allows us to collect only cross progeny that have inheritance of the fluorescent marker from the sire. Three virgin J4 females of *P. exspectatus* were crossed with six fluorescent young-adult males of *P. pacificus* (RS3066). Three fluorescent young-adult F1 males were backcrossed with three hermaphrodites using adults two days after the J4 stage (J4 + 2d) of *P. pacificus* (PS312). Each fluorescent young-adult BC1 male was crossed with 4- to 6-day-old adult hermaphrodites of *P. pacificus* (PS312) to test the fertility of the male. Animals were allowed to mate for six days, and the number of fluorescent hermaphroditic progeny was counted as the number of hatched progeny. After fertility testing, BC1 males were lysed for genotyping. In addition, the presence/absence of hatched progeny of fluorescent J4 BC1 hermaphrodites was tested for six days without crossing (*N* = 25). Note that there were no progeny in any BC1 hermaphrodites in this experiment.

For QTL analysis of hermaphrodite sterility, we crossed *P. pacificus* females with P. e*xspectatus* males. In this crossing scheme, hermaphrodites of *P. pacificus* (PS312) were run out of self-sperm in four days (J4 + 4d) or five days (J4 + 5d) after the J4 stage before crossing. Three J4 + 4d hermaphrodites of *P. pacificus* were crossed with six young-adult males of *P. exspectatus* (RS5522B). One young-adult F1 male was backcrossed with one J4 + 5d hermaphrodite of *P. pacificus*. BC1 J4 hermaphrodites were isolated without males, and their progeny were counted for six days. After fertility testing, BC1 hermaphrodites were lysed for genotyping. To exclude rare self-progeny after five days, BC1 hermaphrodites without male siblings were excluded, and BC1 hermaphrodites that produced more than 20 progeny, >80% of which show normal development, were defined as putative self-progeny. We sequenced the genome of 2 out of 20 putative self-progeny and confirmed that those have no introgressions. Those putative self-progeny were also excluded.

### Genotyping and coverage analysis of BC1

To obtain informative SNPs for genotyping, Illumina sequence reads of the pooled genome of *P. exspectatus*^81^ (SRR646204) were mapped to the *P. pacificus* reference genome using BWA-MEM as described. SNPs were called using samtools mpileup^72^ with -g option and bcftools^71^ (1.8–12) with -m option and filtered to select for SNPs with ≥98% alternative allele and coverage depth = 10–300 as *P. exspectatus*-specific SNPs. Only reads with the base quality ≥ 13 were counted. The region file of the *P. exspectatus*-specific SNPs was produced by the perl script.

Single-worm whole-genome sequencing of BC1 animals was conducted in the same way as for *P. exspectatus* individuals. Sequence reads were mapped to the *P. pacificus* reference, and variant information at sites with the *P. exspectatus*-specific SNPs was revealed as vcf files using samtools mpileup with -r option and the region file. To genotype data with a low coverage depth, we calculated

$$r_{{\mathrm{alt}}} = \frac{{\mathop {\sum}\nolimits_{{\mathrm{site}}} A }}{{\mathop {\sum}\nolimits_{{\mathrm{site}}} {A + R} }},$$

in each sliding window, where *A* and *R* are the number of alternative and that of reference allele at the informative site, respectively; *r*_alt_ is the maximum likelihood estimate for the probability to find an alternative allele at the site. Therefore, homozygosity of the alternative and reference alleles presumably results in *r*_alt_ ≈ 1 and *r*_alt_ ≈ 0, respectively. The *r*_alt_ value of each non-overlapping 100 kb window was fitted with a LOESS smooth line in each chromosome with span equal four per chromosome length (Mb). In the hybrid, we determined genotypes with/without introgression using *r*_alt_ values with the threshold that was empirically decided. In backcrosses with *P. pacificus*, an *r*_alt_ value at the centre of the valley between a peak at 0 and the next peak, 0.1, was set as threshold (Supplementary Fig. 12a). In backcrosses with *P. exspectatus*, an *r*_alt_ value at the centre of the valley between a peak at 1 and the next peak, 0.7, was set as threshold (Supplementary Fig. 12b). Because genotype imputation is difficult with aneuploid animals, we used this method and estimated presence/absence of the introgression instead of genotypes at the sites. Sites with an association between the presence/absence of the introgression and the presence/absence of progeny was considered a QTL. Association was tested using Fisher’s exact test. For multiple-comparison correction, the significant two-tailed *P* value of Fisher’s exact test was determined by permutation test with random sorting of individuals with the presence/absence of progeny (*N* = 1,000; *P* value of the permutation test = 0.01). For confirmation, we also performed ordinary QTL analysis using the R package qtl using ‘binary’ model with error.prob = 0.001. This assumes heterozygosity of the introgressions, in which aneuploidy affects the result. All 100 kb windows were used as genetic marker. Genetic distances between markers were calculated, but the order of the markers was fixed as genomic position. Significant QTLs (*P* < 0.05) were identified with permutation test (*N* = 1,000).

To identify deviation of ploidy of animals, we first examined the coverage depth of F1 hybrids by sequencing the genome of five F1 females and five F1 males of a cross of a *P. exspectatus* female with a *P. pacificus* male, for the BC1 female and BC1 male, respectively. Similarly, we sequenced the genome of five F1 females of a cross of a *P. pacificus* female with a *P. exspectatus* male for the BC1 hermaphrodite. Normalized coverage of 100 kb windows was calculated as the coverage depth at any windows divided by the average coverage depth of the individuals. Then difference in the normalized coverage between BC1 and F1 was calculated by subtraction of the log-scaled normalized coverage of F1 from that of BC1 only using the introgression sites of BC1.

### Recombination-rate analysis

To assess recombination rate in *P. exspectatus*, we generated crossing data between *P. exspectatus* ethyl methanesulfonate mutants. Mutagenesis of J4 animals of *P. exspectatus* (RS5522B) was performed as previously described for *P. pacificus*^82^. For genotyping and recombination analysis, we directly used great-grand-progeny (F3) produced in the following cross-scheme to avoid the purge of homozygous mutations in the case of recessive deleterious mutations. After mutagenesis, motile P0 males and females were crossed as one-to-one pairs (*N* = 40). F1 J4 females were crossed with F1 young-adult males from different siblings, again one to one (*N* = 91). We assumed that F2 animals have heterozygous mutations derived from their F1 dam and sire. One F2 female was crossed with one *P. exspectatus* wild-type male (*N* = 62) and produced F3 animals. F3 females were collected for whole-genome sequencing to identify recombination in F2 female meiosis. The whole genomes of the F1 and F3 animals were sequenced using single-worm whole-genome sequencing. A vcf file for SNPs of each animal was obtained as described for the QTL study. The following analysis was conducted with custom perl or R scripts.

The F3 animals were genotyped in a 500 kb sliding-window approach (*N* = 103). Unique SNPs of single F1 animals (ten males and ten females) were searched for in the genomes of their grand-progeny (F3). If a 500 kb sliding window of an F3 genome contained only SNPs shared with either grandam or grandsire, the genotype of the region was determined to be genotype A or genotype B, respectively. If the region contained SNPs from both grandparents or no SNPs from grandparents, the genotypes of regions were left undetermined (U). We used a 500 kb window because each window includes more than two SNPs on average (the mean number of SNPs is 2.543). Because of the limited number of progeny in each family, the genotypes of multiple families were combined in the analysis. Using the genotype data of 500 Mb sliding windows, linkage analysis was performed with the R/qtl pipeline using crosstype = ‘bc’. Markers with segregation distortion (*P* value of chi-squared test with Bonferroni correction < 0.05) were removed. In addition, individuals with ≤200 valid markers (in total, 330 markers) were removed (*N* = 7). The genetic distance between markers was analysed using est.map function with error.prob = 0.001. Finally, if the logarithm of odds increased by removing one maker or the length of linkage groups shortened more than 10 cM by removing one internal marker, the marker was also removed. We iteratively estimated genetic distance until no marker had to be removed. Using genomic coordinates of valid markers (*N* = 306), the recombination rate was calculated between every neighbouring genetic marker and fitted a spline using LOESS function with span = 0.2 in an R script.

Regions with lower recombination than average recombination across the genome (yellow line in Fig. 3b,c and Extended Data Fig. 7) are defined as the low-recombination region. For the bootstrapping test of the percentage of genes in the low-recombination regions, we first fitted the Marey map plots to a spline using LOESS function with span = 0.2. Second, we calculated the total genetic distance of the low-recombination regions (*P. exspectatus*, 34 cM; *P. pacificus*, 23 cM). Third, we performed the bootstrap sampling with 10,000 iterations. In each iteration, one genomic region with the same genetic distance was randomly picked out of the low-recombination region, and the percentage of the genes was calculated. We used either predicted- or expressed-gene dataset as for the genome statistics in the preceding.

To assess the recombination rate in *P. pacificus*, sequence reads of the previously reported genome of strain RSB001^83^ (ERX1461487) were mapped to the *P. pacificus* reference sequence (PS312), and variants were called. SNPs having >98% alternative alleles with a coverage depth = 10–500 were selected as RSB001-specific SNPs. We mapped the RNA-seq reads of RILs^47^ (PRJEB20517) to the *P. pacificus* reference genome sequence using HISAT2^84^ (version 2.2.1) and called SNP using samtools mpileup. Genotypes of a 100 kb sliding window with the number of RSB001-specific SNP > 10 were determined as homozygote for the RSB001 allele, whereas genotypes of the other window were determined as homozygote of the PS312 allele. We used a threshold of 10 because this is the centre of the valley of the histogram (Supplementary Fig. 12c). Windows with allele frequencies for RSB001 in RILs ≤ 0.25 were removed because these regions may have problems for detection of RSB001-specific SNPs. After the removal of windows, the number of RSB001-specific SNPs was fitted to LOESS spline with span = 0.1. The genotype of each RIL was redetermined by the predicted value of the LOESS spline. Using the genotypes of 100 kb windows, linkage analysis was performed with R/qtl with crosstype = ‘riself’.

To assess the recombination rate between the interspecies genome, BC1 males and BC1 hermaphrodites previously analysed in the QTL study were reanalysed for F1 meiosis of *P. exspectatus* and *P. pacificus* and that of *P. pacificus* and *P. exspectatus*, respectively. Trisomy animals categorized in the coverage analysis were removed. In addition, BC1 animals that indicated a change of genotype at the fusion breakpoint (1 Mb near to 21.05 Mb) were also removed as possible candidates for trisomy. Using the genotypes of 100 kb windows, linkage analysis was performed with R/qtl with crosstype = ‘bc’.

Sites of recombination in BC1 males were detected in the genotypes of the QTL study. The effect of recombination sites on fertility for males was tested using likelihood ratio tests between generalized linear models with glm function and family = ‘binomial’ in an R package, stats. Test model includes recombination sites as an explanatory variable, whereas the null model is the intercept-only model, which has the constraint that the coefficient of the recombination site equals zero.

### Analysis of sequence divergence

To compare the sequence divergence of *P. exspectatus* and *P. pacificus* between loci, we estimated the synonymous substitution rate, dS, of genes. Note that the sequence divergence of some non-coding regions of the chromosome periphery is too high for proper alignment. We used neutral mutations of the coding regions as an indicator of divergence of the sequence. The transcript sequence of one-to-one orthologues of the *P. exspectatus* and *P. pacificus* were aligned with MUSCLE^85^ (version 3.8.31). The dS of each transcript was estimated using the Nei-Gojobori method using codeml of PAML^86^ (4.9j) with runmode = −2. The dS was plotted in the genomic position of *P. pacificus* and fitted a spline using LOESS function with span = 0.2 in an R script.

### Reporting summary

Further information on research design is available in the Nature Portfolio Reporting Summary linked to this article.

## Supplementary information

Supplementary Information Supplementary Note, Figs. 1–14, Tables 1–7 and references.

Reporting Summary

## Data Availability

The genome assembly of *P. exspectatus* is available in the European Nucleotide Archive: https://www.ebi.ac.uk/ena/browser/view/PRJEB46690. The data analysed during the current study are available in a Figshare repository: https://figshare.com/projects/Dataset_of_speciation_genetics_of_P_pacificus_vs_P_exspectatus/132506.

## Code availability

The custom scripts were deposited to a GitHub repository: https://github.com/KYPpa/Ppac_Pexs_Spec/tree/master.
