# Supplementary material for: Chromosome fusions repatterned recombination rate and facilitated reproductive isolation during Pristionchus nematode speciation
Source: Nat Ecol Evol. 2023 Jan 30;7(3):424–39. doi: 10.1038/s41559-022-01980-z (PMC9998273; doi:10.1038/s41559-022-01980-z)
Supplement: Supplementary file 1 — Supplementary Note, Figs. 1–14, Tables 1–7 and references. [file 41559_2022_1980_MOESM1_ESM.pdf]

nature ecology & evolution

Article

https://doi.org/10.1038/s41559-022-01980-z

Chromosome fusions repatterned recombination rate and facilitated reproductive isolation during *Pristionchus* nematode speciation

In the format provided by the authors and unedited

Table of contents

Contents	Page
Supplementary Note	2
Supplementary Fig. 1-14	5
Supplementary Table 1-7	19
Reference	27

Supplementary Note

Reproductive isolation between *P. pacificus* and *P. exspectatus*

In hybrid crosses, we observed strong reduction of progeny numbers relative to pure crosses of the parental species (Fig. 1d). This might be explained by premating isolation, postmating prezygotic isolation and/or hybrid inviability of F1 animals. The finding that all hybrid crosses produced progeny (Fig. 1c) suggests that premating isolation is not strong. However, we cannot exclude the possibility of inefficient mating. The number of progeny with immature morphology in hybrid crosses was low (Fig. 1e), suggesting that hybrid progeny develop normally with regard to their morphology. Sex ratio analysis also indicated no quantitative loss of one sex (Extended Data Fig. 1a). In *Caenorhabditis* nematodes, it is known that gonochoristic species often have sperm that kill the female in interspecific crosses[1]. We found that *P. pacificus* hermaphrodites crossed with *P. exspectatus* males often died in the mating period (8 out of 18 crosses), whereas this happened only rarely in the reciprocal cross (1 out of 17 crosses). Such postmating prezygotic isolation might contribute to the reproductive isolation of the species.

In contrast to these hybrid crosses, we observed large reductions in progeny number in backcrosses or intercrosses of F1 animals (Fig. 1g and Supplementary Fig. 1). This reduction might be explained by hybrid sterility of F1 animals or hybrid inviability of BC1 or F2 animals. The difference in the number of progeny between crosses can be explained by the difference in hybrid sterility between sexes. Given that males are the heterogametic sex in *Pristionchus* species, the stronger female sterility of F1 animals is an exception of Haldane's rule. Even if the sister chromosomes pair properly, F1 female meiosis forms tetravalent chromosomes that are even more abnormal than trivalent chromosomes in F1 male meiosis (Supplementary Fig. 13).

We observed propagation of F1 intercrosses between *P. pacificus* and *P. exspectatus* in the first experiment (2 out of 4 replication, Fig. 1b). However, the F1 intercross did not efficiently produced progeny in the second experiment (Supplementary Fig. 1). This is explained by the number of F1 individuals tested. In the first experiment we used >50 F1 females and males to cross in each replication that produced some viable and fertile hybrids. In contrast, we used individual animals in the second set of experiments.

Chromosome evolution in outgroup nematodes.

Synteny analysis with the chromosome-level assembly of different species of *Caenorhabditis* indicated conserved inter-chromosomal synteny among *Caenorhabditis* species[2-6], suggesting that inter-chromosomal rearrangements are rare in these nematodes. In contrast, recent reports of chromosome-level assemblies of outgroup species of *Caenorhabditis* suggest more variation of karyotypes in nematodes[7-13]. Tandonnet and her colleagues

conducted a meta-analysis of synteny of nematodes[9] that was recently updated[13]. They identified chromosome fusions and fissions throughout nematode evolution, which can be explained by rearrangements between seven conserved chromosomal elements that are defined by sets of conserved genes. The seven elements were designated as 'Nigon' elements[9], which are analogous to Müller elements in *Drosophila*. *P. pacificus* ChrV, ChrII, ChrIII, ChrIV, ChrIL, ChrIR and ChrX are corresponding to Nigon elements, NigonA, NigonB, NigonC, NigonD, NigonE, NigonN and NigonX, respectively[13]. Here, we use the original definition of Nigon elements[9]. Although Foster and his colleagues used a different definition[10], Gonzalez de la Rosa and his colleagues justified the original definition using systematic comparison of nematode genome[13].

Chromosome evolution across nematodes is summarized in Supplementary Fig. 14. The chromosome fusions of *P. pacificus* and *P. exspectatus* are corresponding to the fusion between NigonE and NigonN and fusion between NigonN and NigonX, respectively. The *Caenorhabditis* ancestor also independently underwent the fusion between NigonN and NigonX. Fused Nigon elements were highly rearranged after the original fusion events, *i. e.* the *Caenorhabditis* X chromosome (stripe patterns in Supplementary Fig. 14). However, several chromosome fusions in other nematode lineages lack rearrangements between the fused Nigon elements, suggesting these fusions are likely very recent, such as in *P. pacificus*. For example, while an old fusion between NigonD and NigonX was followed by rearrangements between in the common ancestor of *Brugia malayi* and *Onchocerca volvulus*, the X chromosome was subsequently fused to NigonE and NigonN in the *B. malayi* and *O. volvulus* lineage, respectively, but did not undergo rearrangements. In contrast, the lineage of some nematodes, *Auanema rhodensis*, *Strongyloides ratti*, and *Ascaris suum* underwent complicated rearrangements.

The number of events of chromosome evolution might be underestimated due to small taxon sampling, as independent fusions between the same combination of elements might go unnoticed. One clear example for this is the fusion between NigonN and NigonX in the lineage of *Haemonchus contortus*, which is related to *C. elegans*. Synteny analysis between *H. contortus* and *C. elegans* indicated homologous inter-chromosomal synteny and their karyotypes were first thought to be highly conserved[12]. However, by synteny analyses with the other species, Gonzalez de la Rosa and his colleagues reported two lines of evidence that the fusion of NigonN and NigonX in the *H. contortus* lineage is independent from *C. elegans*[13]. First, the chromosome-level assembly of *A. rhodensis* and *Oscheius tipulae*, both of which are more closely related to *H. contortus* than *C. elegans*, indicated that the NigonN and NigonX are corresponding to different chromosomes. Second, the draft assembly of *Diploscapter coronatus*, which is more closely related to *C. elegans* than *H. contortus*, indicates that long contigs include NigonX, but not NigonN. Both results suggest that the common ancestor of *H. contortus* and *C. elegans* had no fusion between NigonN and NigonX and that these fusions

occurred independently in both lineages. Given that *P. exspectatus* also experienced a fusion between NigonN and NigonX, some combinations of Nigon elements might have an evolutionary trend to fuse frequently, which makes underestimation of the number of evolutionary events in parsimonious estimations. More data of different species will improve our understanding of chromosome evolution of nematodes and can reveal the generality of findings of the present study.

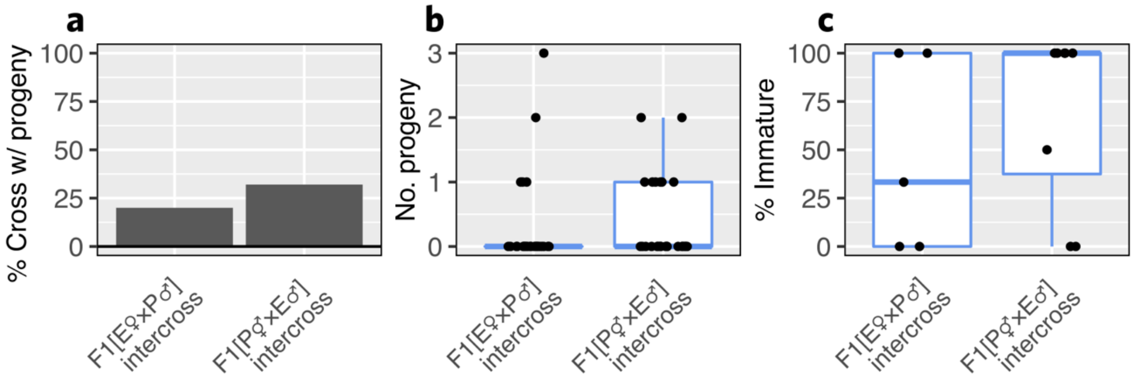

Supplementary Fig. 1 | F1 intercrosses between *P. exspectatus* and *P. pacificus*. The proportion of crosses with progeny (**a**), the number of the progeny (**b**) and the proportion of immature progeny (**c**) are shown. F1[E♀×P♂], F1 produced by a cross between a *P. exspectatus* female and a *P. pacificus* male. F1[P⚥×E♂], F1 animals produced by a cross between a *P. pacificus* hermaphrodite and a *P. exspectatus* male. For the number of progeny and proportion of immature progeny, all replicates are shown as jitter plot. The box plot indicates: upper whisker, the largest data point less than the third quartile+1.5×interquartile range; upper bound, the third quartile; center line, median; lower bound, the first quartile; lower whisker, the smallest data point more than the first quartile−1.5×interquartile range. The sample number is shown in Supplementary Table 1.

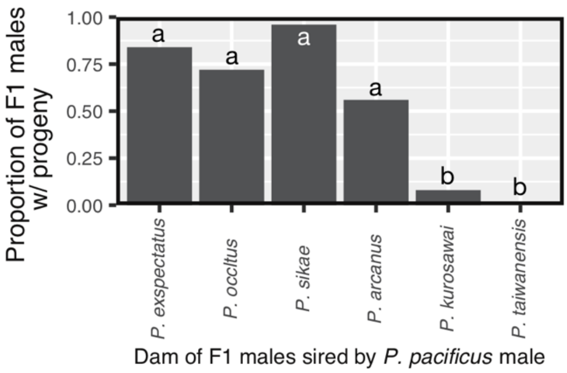

Supplementary Fig. 2 | Backcrosses of species hybrids. F1 males produced by the crosses between *P. pacificus* males and females of related species were backcrossed with females of the same species as their dam. The proportion of the cross with BC1 progeny was investigated in crosses between a single female and a single male (*N*=25 each). The letter above or on the bar indicates statistically different groups. Any pair from the different group shows significant difference in the proportion using Fisher's exact test (two-tailed $p < 0.05$).

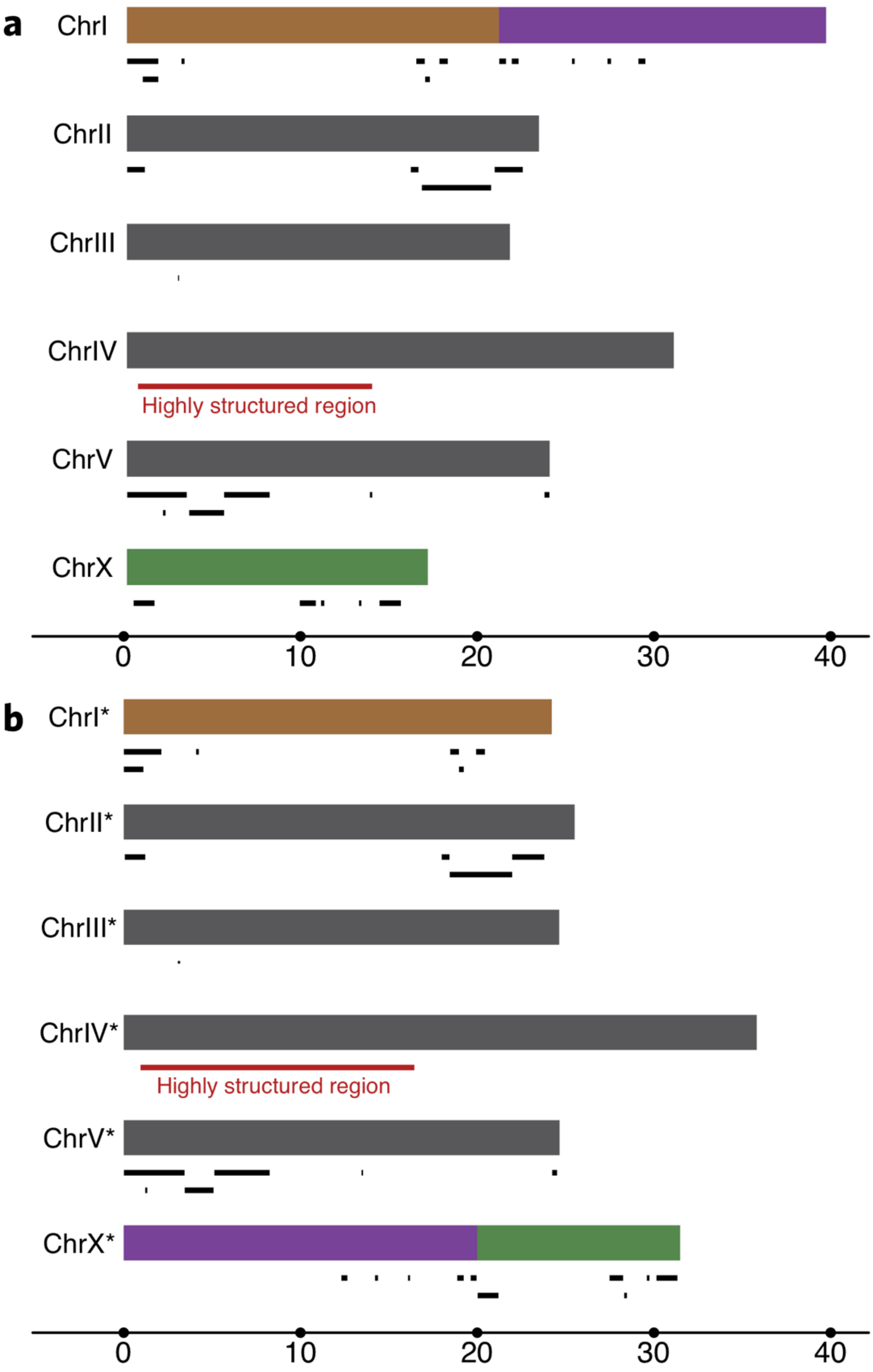

Supplementary Fig. 3 | Position of >100kb intrachromosomal rearrangements between *P. pacificus* and *P. exspectatus*. The inversions in coordinates of *P. pacificus* (**a**) and *P. exspectatus* (**b**) are shown. The genomic regions of ChrIL, ChrIR, ChrX and the other chromosomes are represented in orange, purple, green and grey rectangles, respectively. Black lines indicate the position of inversions. Red lines indicate the highly structured region.

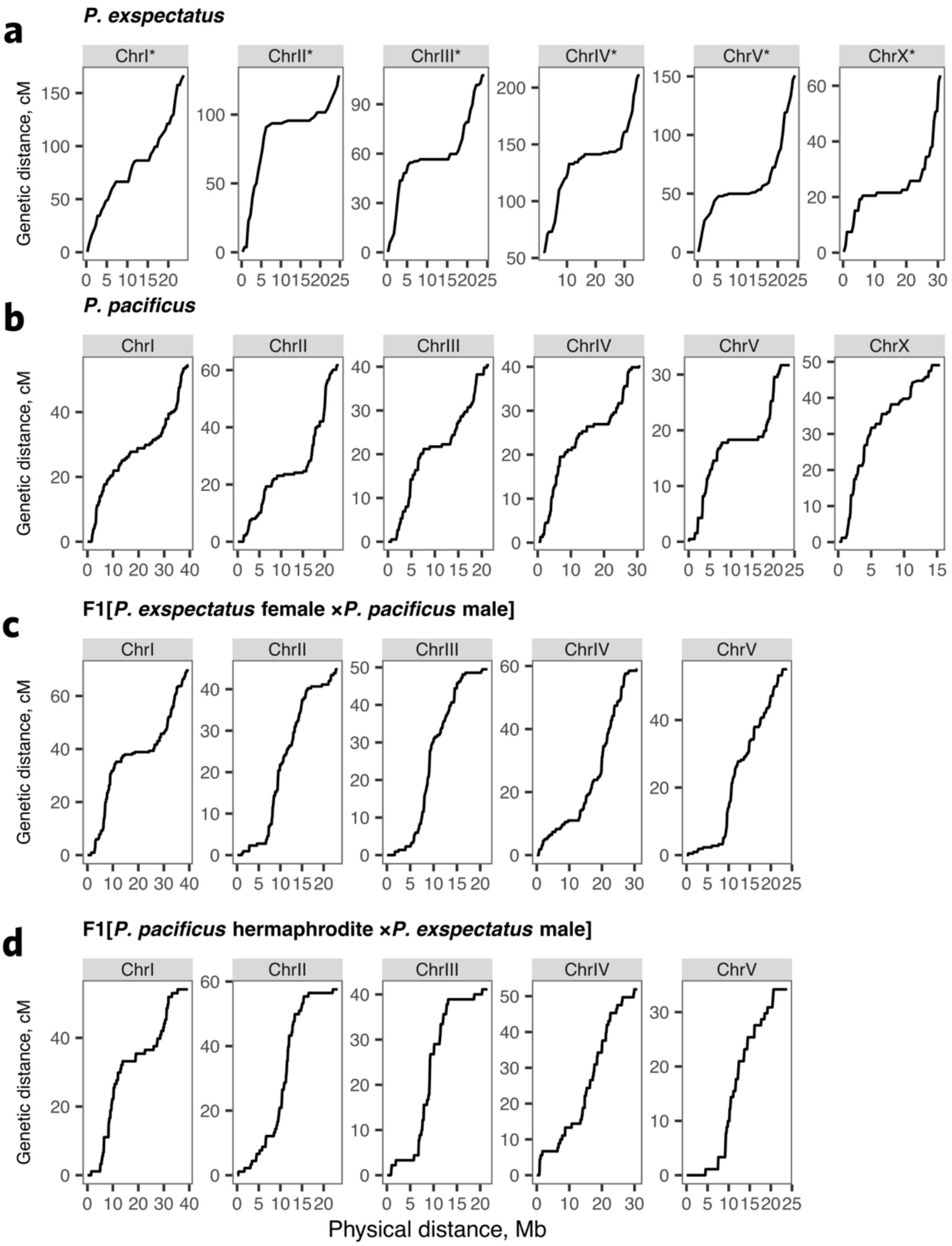

Supplementary Fig. 4 | Marey map. The physical distance (Mb) in the genome sequence and genetic distance (cM) calculated by linkage analysis from the genetic marker at the left-end of the chromosome are compared.

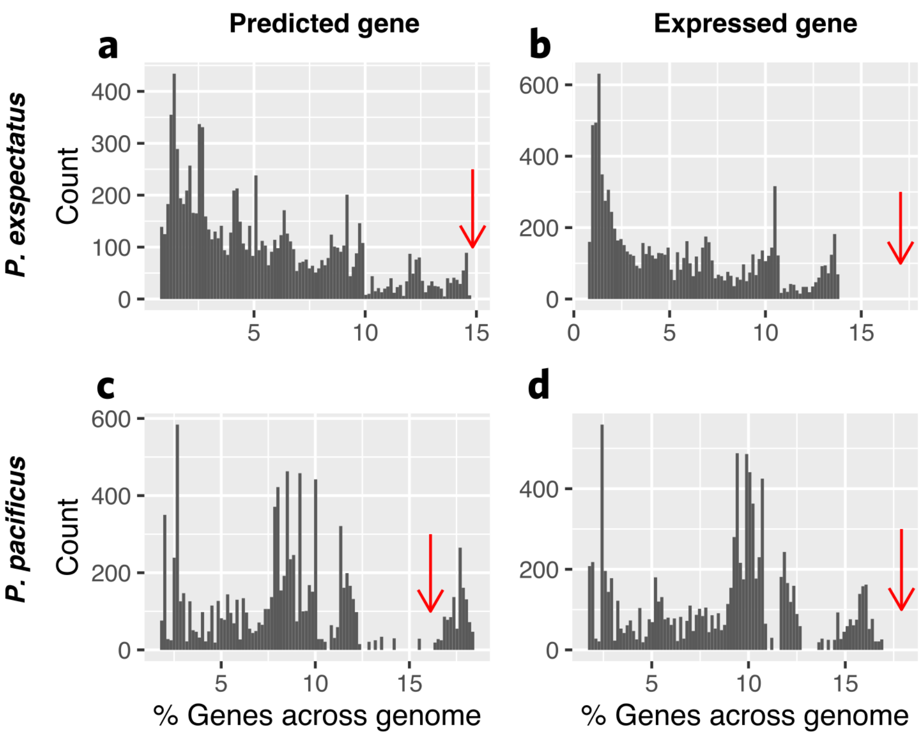

Supplementary Fig. 5 | Bootstrap test of the percentage of genes in the low recombination region around the site of chromosome fusions in *P. exspectatus* (a and b) and *P. pacificus* (c and d). Genomic regions with the same genomic distance as the low recombination regions of the chromosome fusions were randomly sampled out of the low recombination regions and counted along with the percentage of genes (N = 10,000). The predicted genes (**a** and **c**) or expressed genes of the young adults (**b** and **d**) were used. Silenced unfunctional genes are not contained in the expressed genes while the expressed genes may be conditional. The percentage of genes in the low-recombination regions around the chromosome fusions are shown as the red arrows. The percentage of genes in the low recombination regions is significantly high in **a**, **b** and **d** (one-tailed $p<0.0001$) but not in **c** (one-tailed $p>0.05$). The bin number in the figure is 100.

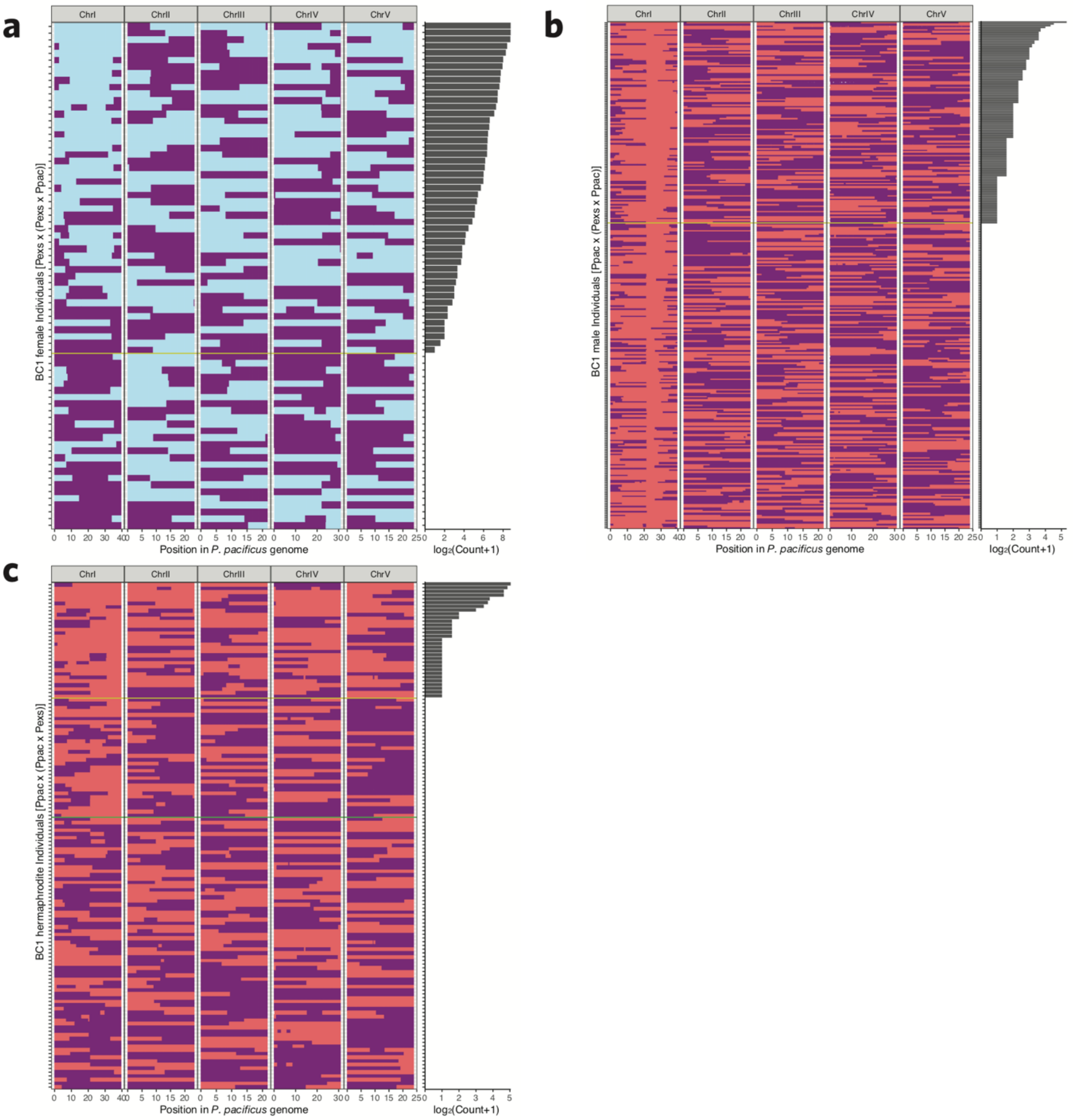

Supplementary Fig. 6 | Introgression map of BC1 female (a), male (b) and hermaphrodite (c) hybrids. Each row shows the genotypes of each individual. Dark purple indicates regions containing introgression allele. Light blue or light red indicate *P. exspectatus* or *P. pacificus* background, respectively. The number of progeny is shown in the bar plot. The yellow line indicates the boundary of presence/absence of progeny. The green line indicates the boundary of presence/absence of eggs in BC1 hermaphrodites.

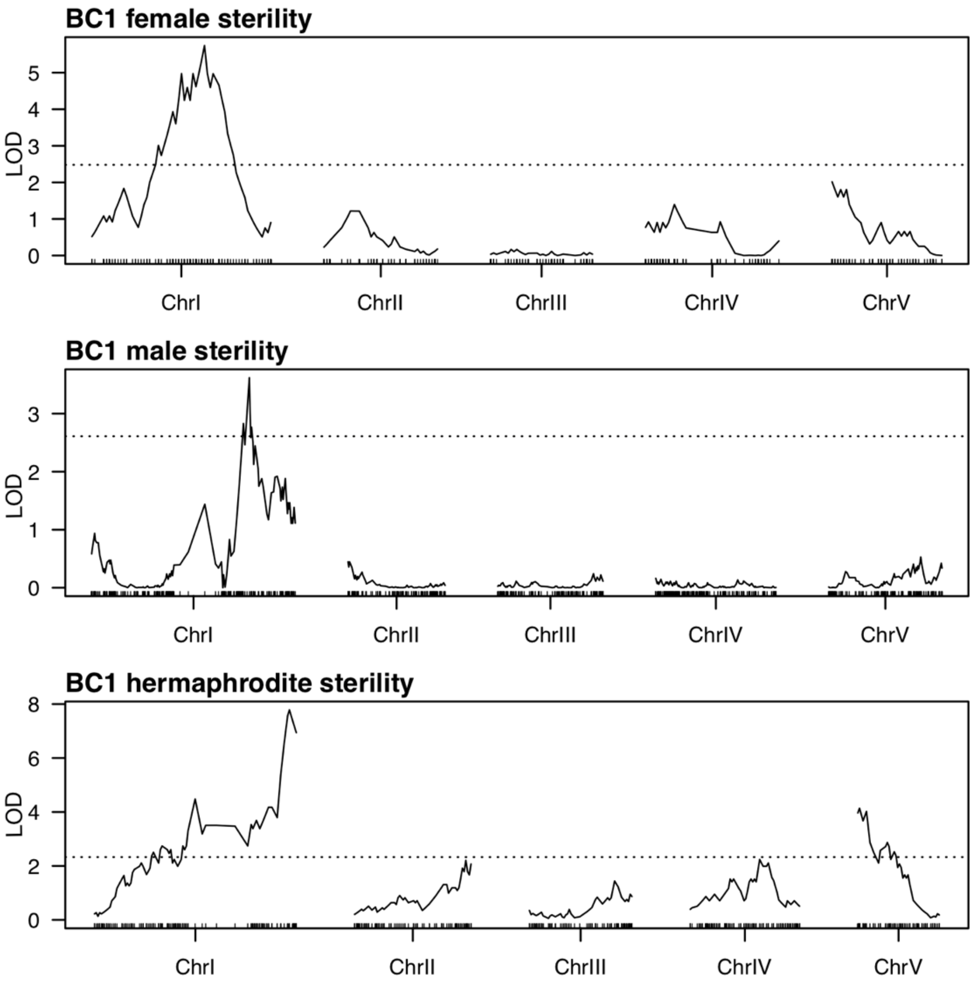

Supplementary Fig. 7 | Results of QTL analysis using R/qtl. Logarithm of odds (LOD) score is shown on the *Y*-axis. The significant threshold of LOD was calculated by permutation tests (*N* = 1000, significant level = 0.05) The bottom ticks indicate the position of genetic markers, which is the genotype of 100kb sliding window of the genome.

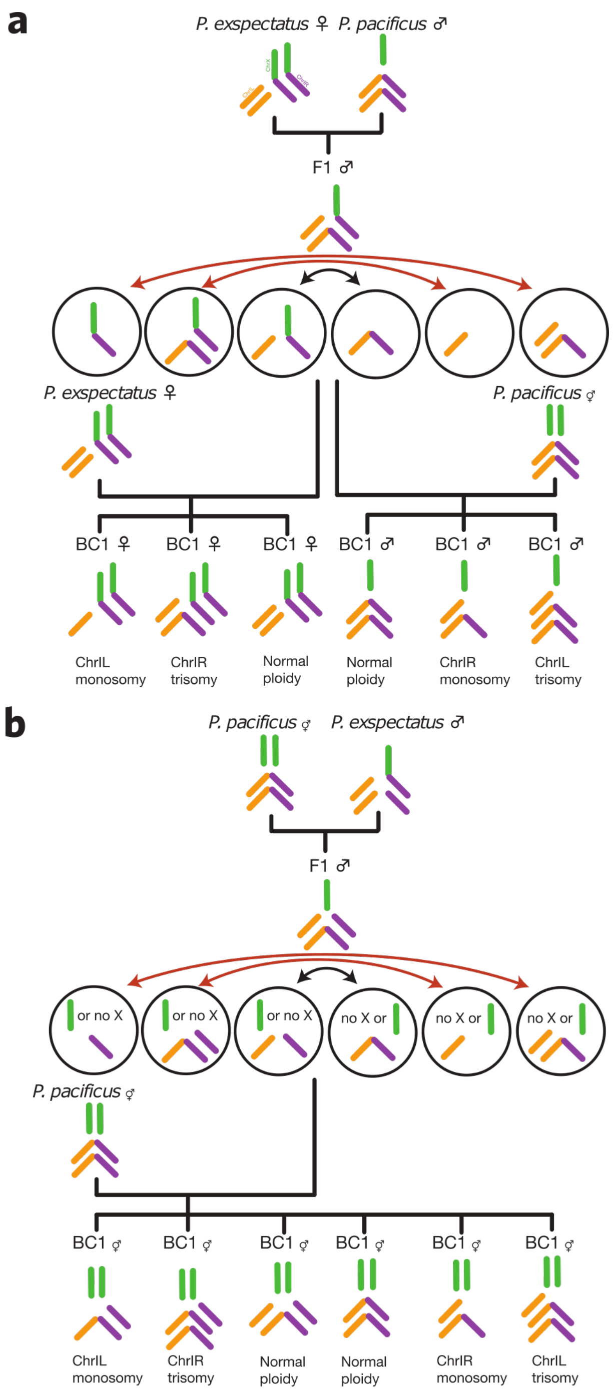

Supplementary Fig. 8 | Expected karyotypes of BC1 animals with aneuploidy. a, Expected karyotypes of the BC1 females and males after a cross between a *P. exspectatus* female and a *P. pacificus* male. **b**, Expected karyotypes of the BC1 hermaphrodites after a cross between a *P. pacificus* hermaphrodite and a *P. exspectatus* male. Black and red arrows indicate balanced and unbalanced segregation in F1 meiosis, respectively. Black circles show the haplotypes of sperm cells.

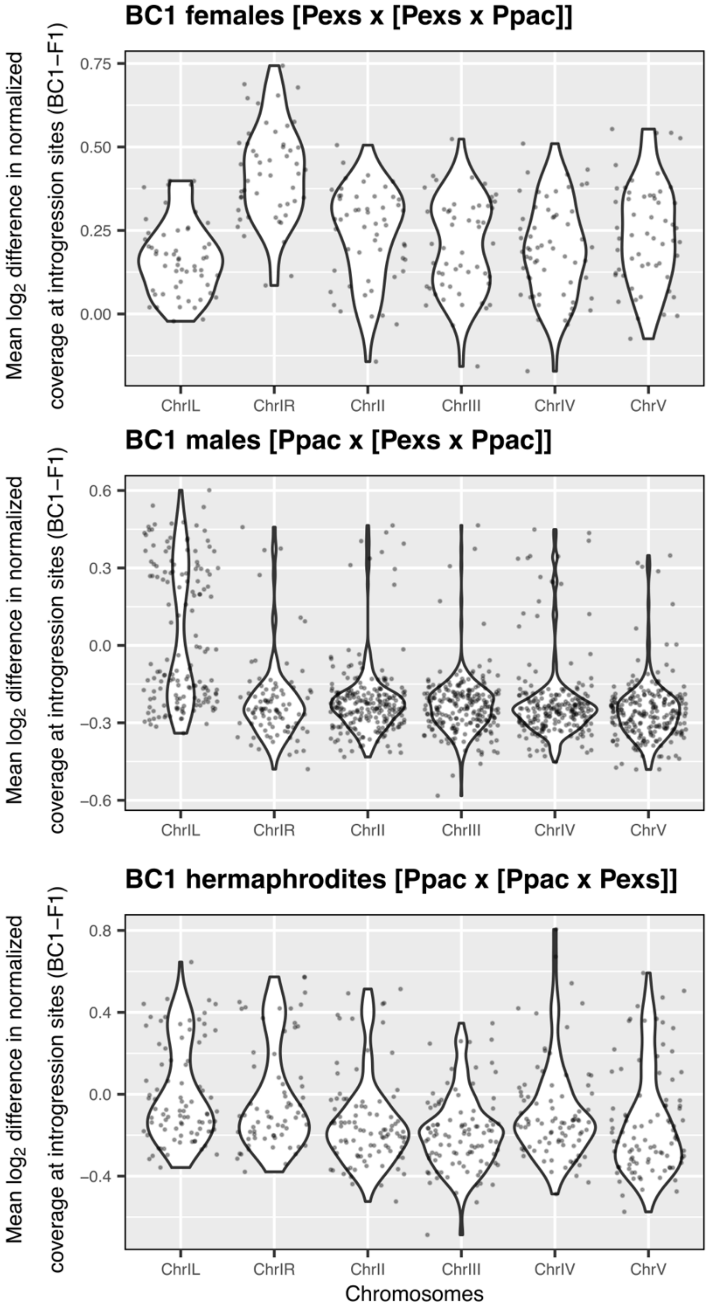

Supplementary Fig. 9 | Violin plot of increased normalized coverage of genome sequences of BC1 animals. Because the mapping rate is different between the *P. exspectatus* and *P. pacificus* alleles to the *P. pacificus* reference, only introgression sites, which are heterozygous in diploid, were used to analyze the coverage depth. The coverage depth of introgression sites of BC1 was first log_2-transformed and normalized by average coverage depth and subtracted by the normalized coverage depth of the same site of F1 hybrids. The plots show the mean of the log_2 difference in the coverage depth at introgression sites in the chromosomes of single individuals.

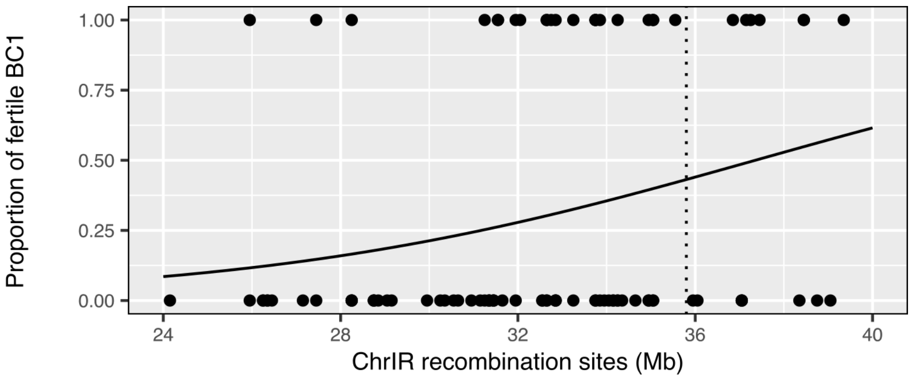

Supplementary Fig. 10 | Predicted proportion of fertile BC1 along with recombination sites by a generalized linear model. The predicted logistic regression is shown as the solid line. The dot line indicates the peak of recombination rate of *P. pacificus* as Fig. 5c. The plots indicate recombination sites of fertile (top) and sterile (bottom) individuals.

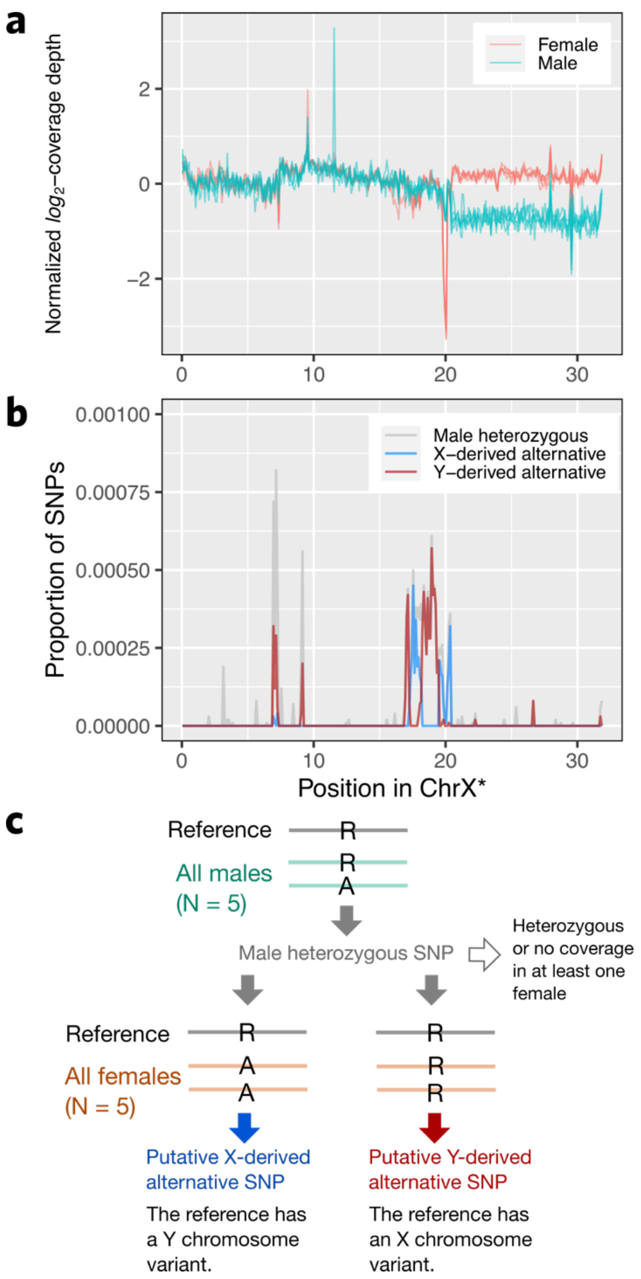

Supplementary Fig. 11 | Y-sequence contamination in the assembly before manual fixation. a, Female-specific reduction of coverage of the X chromosome of *P. exspectatus*. Coverage depth of each 100kb sliding window is normalized by average coverage and $\log_2$-transformed. Each line indicates the value of single individuals. **b**, X-derived alternative SNPs around the fusion breakpoint. The proportion of male-specific SNPs, X-derived alternative SNPs and Y-derived alternative SNPs was calculated in each 100kb sliding window. **c**, Method for identification of male heterozygous SNP, X-derived alternative SNPs and Y-derived alternative SNPs. R and A represent reference and alternative allele, respectively. The results for the same analysis of the final assembly (after the manual fixation) are shown in Extended Data Fig. 8.

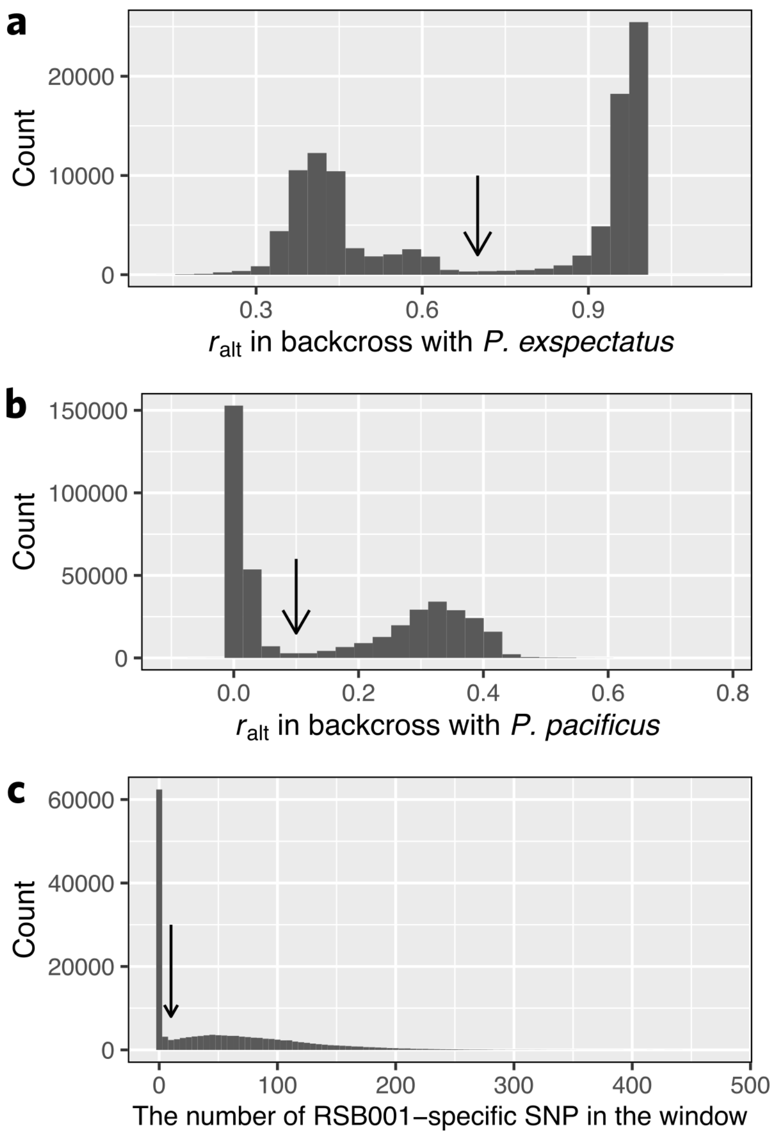

Supplementary Figure 12 | Histogram of values to determine the threshold for genotyping. The thresholds were set at the bin with minimum counts between 1 and the next peak (**a**) or between 0 and the next peak (**b** and **c**) shown as arrows.

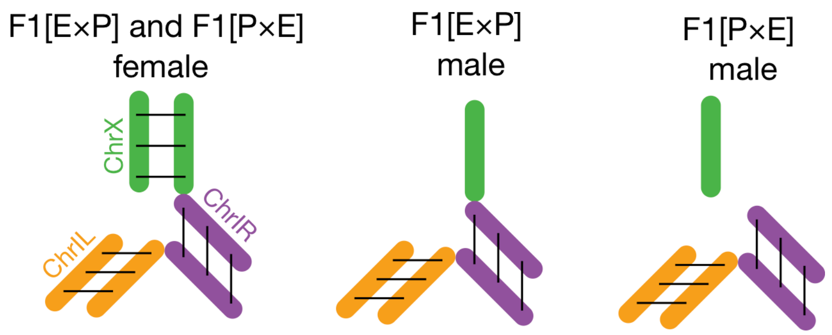

Supplementary Figure 13 | Putative abnormal chromosome pairing in F1 meiosis. Potential regions of synapsis are shown in black lines.

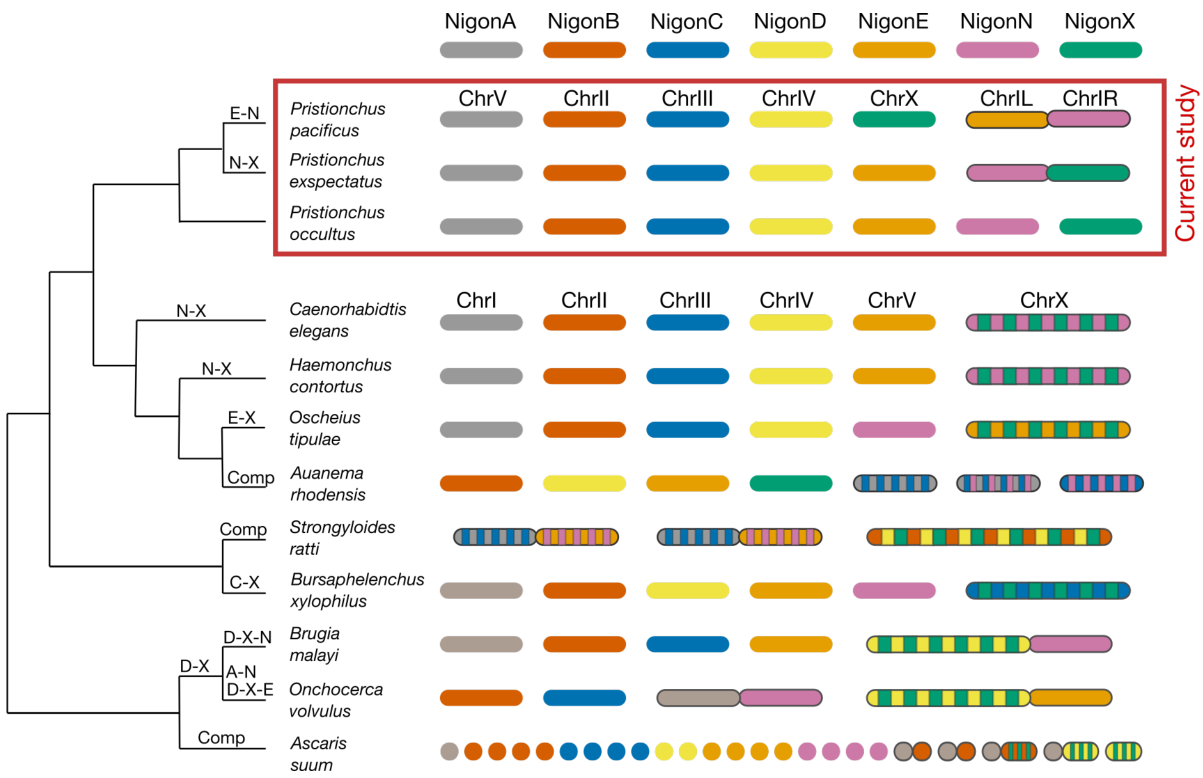

Supplementary Figure 14 | Chromosome evolution across Rhabditida. Chromosomes of each species are shown as bars with colors corresponding to Nigon elements. The black-outlined bars represent fused chromosomes. The stripped color indicates fused Nigon elements having rearrangements with each other. The identification of Nigon elements and estimation of their fusions of out-group species is according to ref.[13]. The phylogeny of the outgroup species is according to ref.[14]. Fusion events between Nigon elements are shown on the branches but the complex rearrangements are shown as "Comp". The chromosome names of *P. pacificus* and *C. elegans* are shown. Note that the order and orientation of chromosomes can be different from the genome assembly of each species.

Supplementary Table 1 | Sample number of quantitative reproduction test

Experiment[1]	Dam[2]	Sire[2, 3]	No. of crosses[4]	No. of crosses with progeny[5]	No of crosses with mature progeny[6]
A	*P. pacificus*	*P. pacificus*	18	18	18
	P. exspectatus	*P. pacificus*	17	16	16
	P. pacificus	*P. exspectatus*	18	17	17
	P. exspectatus	*P. exspectatus*	17	17	17
B	F1[ExP]	-	19	0	0
	F1[ExP]	*P. pacificus*	17	1	1
	F1[ExP]	*P. exspectatus*	18	5	5
	P. exspectatus	F1[ExP]	30	24	24
	P. exspectatus	*P. exspectatus*	20	19	19
C	F1[PxE]	-	30	0	0
	F1[PxE]	*P. pacificus*	20	11	6
	F1[PxE]	*P. exspectatus*	20	18	13
	P. exspectatus	F1[PxE]	20	17	17
	P. exspectatus	*P. exspectatus*	20	19	19
D	F1[ExP]	F1[ExP]	25	5	3
	F1[PxE]	F1[PxE]	25	8	3

[1]A, hybrid crosses between *P. pacificus* and *P. exspectatus*; B, backcrosses with F1 animals produced by a cross between a *P. exspectatus* female and a *P. pacificus* male; C, backcrosses with F1 animals produced by a cross between a *P. pacificus* hermaphrodite and a *P. exspectatus* male; D, intercross of F1 hybrids.

[2] F1[ExP], F1 produced by a cross between a *P. exspectatus* female and *P. pacificus* male; F1[PxE], F1 produced by a cross between a *P. pacificus* hermaphrodite and *P. exspectatus* male.

[3], hyphen indicates that no males were used to test self-fertilization.

[4], the sample number of panels c, d, f, g, i and j of Fig. 2 and panel a and b of Supplementary Fig. 1

[5], the sample number of panels e, h, and k of Fig. 2 and panel c of Supplementary Fig. 1

[6], the sample number of Extended Data Fig. 1

Supplementary Table 2 | Statistics of genome assembly and gene annotations

**Species**	*P. exspectatus*		*P. pacificus*
**Version of genome sequence**	Present study	Prabh *et al.*, 2018[15]	Rödelsperger *et al.*, 2017[8]
**Version of gene annotation**	Present study	Rödelsperger, 2021[16]	Athanasouli *et al.*, 2020[17]
**Total length**	169.6Mb	177.7Mb	158.5Mb
**Contig Number**	24	4412	47
**N50**	25.5Mb	0.142Mb	21.7Mb
**% of the largest 6 contigs**	98.1%	3.01%	98.7%
**Total gene number**	31,021	35,595	28,896
**BUSCO odb9 C/D/F/M (%)[1]**	90/3/4/4	85/7/5/4	97/2/2/0

[1]C, complete as a single-copy gene; D, duplicated; F, fragmented; M, missing.

Supplementary Table 3 | The number of crosses for QTL analysis

Test	Generation[1]	Female or hermaphrodites			Male			No. cross	Analyzed samples[3]
		Species	Strain	No.	Species	Strain[2]	No.		
QTL for female sterility	1st	*P. exspectatus*	RS5522B	3	*P. pacificus*	PS312	6	3	
	2nd	*P. exspectatus*	RS5522B	1	F1 hybrid	-	1	30	
	3rd (T)	BC1 hybrid	-	1	*P. exspectatus*	RS5522B	1	75	N=75
QTL for male sterility	1st	*P. exspectatus*	RS5522B	3	*P. pacificus*	RS3066 [RFP+]	6	2	
	2nd	*P. pacificus*	PS312	3	F1 hybrid	[RFP+]	3	49	
	3rd (T)	*P. pacificus*	PS312	1	BC1 hybrid	[RFP+]	1	312	N=291
QTL for hermaphrodite sterility	1st	*P. pacificus*	PS312	3	*P. exspectatus*	RS5522B	6	8	
	2nd	*P. pacificus*	PS312	1	F1 hybrid	-	1	171	
	3rd (T)	BC1 hybrid	-	1	No male	-	0	183	N=136

[1]T, cross for the test of BC1 fertility.

[2] [RFP+], selected individuals with the reporter genes, RFP,

[3] The number of analyzed samples is different from the number of crosses because of the death of animals during the test, low quality of WGS library and removal of self-progeny.

Supplementary Table 4 | The number of trisomy animals of expected chromosomes in BC1

Cross[1]	Sex	Expected chromosome	Threshold	*N*	No. of trisomy of the expected chromosome	Median no. of. trisomy of the other chromosomes	median *p*-value[2]
E × (E × P)	Female	ChrIR	0.35	75	34 [45%]	11 [18.4%]	**$p = 6.9 \times 10^{-5}$**
P × (E × P)	Male	ChrIL	0	291	64 [22%]	8 [2.7%]	**$p = 2.8 \times 10^{-13}$**
P × (P × E)	Hermaphrodite	ChrIL	0.25	136	19 [14%]	7.5 [5.5%]	**$p = 0.031$**
		ChrIR	0.25	136	14 [10%]	7.5 [5.5%]	$p = 0.21$

[1], E or P from left to right indicates species of dam, grandam and grandsire. E, *P. exspectatus*; P, *P. pacificus*.
[2], the median of two-tailed *p*-value of Fisher's exact test of Trisomy (Yes or No) *vs.* expected/other chromosomes

Supplementary Table 5 | Association of trisomy with fertility of BC1 animals

Cross[1]	Sex	Region	Trisomy	Total no.	Progeny		
					Presence [%]	Absence [%]	*p*-value[2]
E × (E × P)	Female	ChrIR	Yes	34	15 [44%]	19 [57%]	***p* = 0.00061**
			No	41	34 [83%]	7 [15%]	
P × (E × P)	Male	ChrIL	Yes	64	23 [36%]	41 [64%]	*p* = 0.56
			No	227	93 [41%]	134 [59%]	
P × (P × E)	Hermaphrodite	ChrIL	Yes	19	6 [32%]	13 [68%]	*p* = 0.38
			No	117	25 [21%]	92 [79%]	
		ChrIR	Yes	14	2 [14%]	12 [86%]	*p* = 0.52
			No	122	29 [24%]	93 [76%]	

[1], E or P from left to right indicates species of dam, grandam and grandsire. E, *P. exspectatus*; P, *P. pacificus*.

[2], two-tailed *p*-value of Fisher's exact test of Trisomy (Yes or No) *vs.* Presence/absence of progeny

Supplementary Table 6 | The number of individuals in the first crossing experiment between *P. exspectatus* and *P. pacificus*.

Replication	Stages	Sex[1]	No. of individuals					
			Day 5[2]	Day 10	Day 15	Day 20	Day 25	Day 30
R1	Adult	H or F	39	26	39	34	27	27
		M	93	72	81	79	49	46
	J4 juvenile	H or F	8	1	1	0	0	1
		M	28	2	4	2	1	3
	J2-J3 juvenile		77	9	10	1	4	7
	**Total**		**245**	**110**	**135**	**116**	**81**	**84**
	**Sex ratio**		**77%**	**74%**	**69%**	**70%**	**65%**	**65%**
R2	Adult	H or F	70	48	74	59	71	33
		M	54	55	55	54	65	34
	J4 juvenile	H or F	27	2	0	2	1	1
		M	61	4	5	3	1	2
	J2-J3 juvenile		143	19	13	12	7	6
	**Total**		**355**	**128**	**147**	**130**	**145**	**76**
	**Sex ratio**		**67%**	**56%**	**45%**	**50%**	**49%**	**53%**
R3	Adult	H or F	24	15	21	14	10	10
		M	20	16	13	8	11	4
	J4 juvenile	H or F	3	1	0	0	0	0
		M	7	1	0	0	0	0
	J2-J3 juvenile		41	11	3	2	0	0
	**Total**		**95**	**44**	**37**	**24**	**21**	**14**
	**Sex ratio**		**56%**	**55%**	**38%**	**36%**	**52%**	**29%**
R4	Adult	H or F	92	78	84	83	55	33
		M	103	132	131	71	21	10
	J4 juvenile	H or F	4	0	0	1	0	0
		M	78	5	1	0	1	1
	J2-J3 juvenile		47	14	9	10	6	1
	**Total**		**324**	**229**	**225**	**165**	**83**	**45**
	**Sex ratio**		**67%**	**64%**	**61%**	**46%**	**29%**	**25%**

(continued)

[1], H or F, hermaphrodite or female; M, male. [2], the number of individuals reduces after Day 5 because only 25% of individuals were transferred between Day 5 and Day 10.

Supplementary Table 6 (continued)

Replication	Stages	Sex[1]	No. of individuals					
			Day 5[2]	Day 10	Day 15	Day 20	Day 25	Day 30
R5	Adult	H or F	104	72	76	47	37	17
		M	65	56	62	53	27	10
	J4 juvenile	H or F	43	2	1	1	0	0
		M	60	1	0	1	0	0
	J2-J3 juvenile		131	14	7	0	0	0
	**Total**		**403**	**145**	**146**	**102**	**64**	**27**
	**Sex ratio**		**62%**	**45%**	**45%**	**54%**	**42%**	**37%**
R6	Adult	H or F	71	49	46	30	20	8
		M	29	24	22	20	16	28
	J4 juvenile	H or F	30	0	0	1	0	0
		M	34	0	0	0	3	0
	J2-J3 juvenile		123	2	4	1	1	0
	**Total**		**287**	**75**	**72**	**52**	**40**	**36**
	**Sex ratio**		**57%**	**33%**	**32%**	**41%**	**49%**	**78%**
R7	Adult	H or F	83	38	53	54	45	26
		M	91	46	36	45	27	11
	J4 juvenile	H or F	8	1	2	1	2	1
		M	37	2	2	0	2	1
	J2-J3 juvenile		35	7	5	6	12	9
	**Total**		**254**	**94**	**98**	**106**	**88**	**48**
	**Sex ratio**		**62%**	**56%**	**43%**	**46%**	**41%**	**33%**
R8	Adult	H or F	28	21	18	11	7	6
		M	18	15	18	13	3	2
	J4 juvenile	H or F	8	0	0	0	0	0
		M	20	0	0	0	0	0
	J2-J3 juvenile		82	3	2	0	0	0
	**Total**		**156**	**39**	**38**	**24**	**10**	**8**
	**Sex ratio**		**62%**	**42%**	**50%**	**54%**	**30%**	**25%**

[1], H or F, hermaphrodite or female; M, male. [2], the number of individuals reduces after Day 5 because only 25% of individuals were transferred between Day 5 and Day 10.

Supplementary Table 7 | FISH probes

	Probe Name	Modification	Sequence
ChrIL	PpacChrILpA01	DY415	CCAGTGCTTCCACAACCGAA
	PpacChrILpA02	DY415	CCCTCGACTAGCGCGTCTAC
	PpacChrILpA03	DY415	CACCGAGCCAAGCACCTCTG
	PpacChrILpA04	DY415	CTTCCACAACCGAGCCCTCG
	PpacChrILpA05	DY415	ACCAGTGCTTCCACAACCGA
ChrIR	PpacChrIRpA01	Fluorescein	GTGCTTCCCTCGGTGCTCATC
	PpacChrIRpA02	Fluorescein	GAGAATTCAGTCGATGATGGAG
	PpacChrIRpA03	Fluorescein	TTCGACCATCGAGCCCACCTCTAC
ChrX	PpacChrXpA01	Cy3	ACATGTCGTCCTCAATACATC
	PpacChrXpA02	Cy3	GTTTCTTGACGTGTATCTTGA
	PpacChrXpA03	Cy3	ACACAATAGGCACATACGTGA
	PpacChrXpA04	Cy3	ACCACTGAGCTAACAGGGCTC
	PpacChrXpA05	Cy3	GTAGAGCATCAGACTTTTAAT
	PpacChrXpA06	Cy3	TCGAACCACCGTCCTCCAGAT
	PpacChrXpA07	Cy3	GATTCCACTACGAGGCTTAAC

References

1. Ting, J. J. et al. Intense sperm-mediated sexual conflict promotes reproductive isolation in *Caenorhabditis* nematodes. *PLoS Biol.* **12**, e1001915 (2014).
2. Yin, D. et al. Rapid genome shrinkage in a self-fertile nematode reveals sperm competition proteins. *Science* **359**, 55-61 (2018).
3. Stevens, L. et al. The genome of *Caenorhabditis bovis*. *Curr. Biol.* **30**, 1023-1031. e4 (2020).
4. Teterina, A. A., Willis, J. H. & Phillips, P. C. Chromosome-level assembly of the *Caenorhabditis remanei* genome reveals conserved patterns of nematode genome organization. *Genetics* **214**, 769-780 (2020).
5. Noble, L. M. et al. Selfing is the safest sex for *Caenorhabditis tropicalis*. *eLife* **10**, e62587 (2021).
6. Stevens, L. et al. Chromosome-level reference genomes for two strains of *Caenorhabditis briggsae*: an improved platform for comparative genomics. *Genome Biol. Evol.* **14**, evac042 (2022).
7. Cotton, J. A. et al. The genome of *Onchocerca volvulus*, agent of river blindness. *Nat. Microbiol.* **2**, 1-12 (2016).
8. Rödelsperger, C. et al. Single-molecule sequencing reveals the chromosome-scale genomic architecture of the nematode model organism *Pristionchus pacificus*. *Cell Rep.* **21**, 834-844 (2017).
9. Tandonnet, S. et al. Chromosome-wide evolution and sex determination in the three-sexed nematode *Auanema rhodensis*. *G3: Genes Genomes Genet.* **9**, 1211-1230 (2019).
10. Foster, J. M. et al. Sex chromosome evolution in parasitic nematodes of humans. *Nat. Commun.* **11**, 1-12 (2020).
11. Wang, J. et al. Comprehensive chromosome end remodeling during programmed DNA elimination. *Curr. Biol.* **30**, 3397-3413. e4 (2020).
12. Doyle, S. R. et al. Genomic and transcriptomic variation defines the chromosome-scale assembly of *Haemonchus contortus*, a model gastrointestinal worm. *Commun. Biol.* **3**, 1-16 (2020).
13. Gonzalez de la Rosa, P. M. et al. A telomere-to-telomere assembly of *Oscheius tipulae* and the evolution of rhabditid nematode chromosomes. *G3: Genes Genomes Genet.* **11**, jkaa020 (2021).
14. Blaxter, M. & Koutsovoulos, G. The evolution of parasitism in Nematoda. *Parasitol.* **142**, S26-S39 (2015).
15. Prabh, N. et al. Deep taxon sampling reveals the evolutionary dynamics of novel gene families in Pristionchus nematodes. *Genome Res.* **28**, 1664-1674 (2018).
16. Rödelsperger, C. The community-curated *Pristionchus pacificus* genome facilitates automated gene annotation improvement in related nematodes. *BMC Genomics* **22**, 1-12 (2021).
17. Athanasouli, M. et al. Comparative genomics and community curation further improve gene annotations in the nematode *Pristionchus pacificus*. *BMC genomics* **21**, 1-9 (2020).
