# Supplementary material for: Chromosome fusions repatterned recombination rate and facilitated reproductive isolation during Pristionchus nematode speciation
Source: Nat Ecol Evol. 2023 Jan 30;7(3):424–39. doi: 10.1038/s41559-022-01980-z (PMC9998273; doi:10.1038/s41559-022-01980-z)
Supplement: Supplementary file 2 — Reporting Summary [file 41559_2022_1980_MOESM2_ESM.pdf]

nature portfolio

Corresponding author(s): Ralf J. Sommer and Kohta Yoshida

Last updated by author(s): Dec 20, 2022

Reporting Summary

Nature Portfolio wishes to improve the reproducibility of the work that we publish. This form provides structure for consistency and transparency in reporting. For further information on Nature Portfolio policies, see our Editorial Policies and the Editorial Policy Checklist.

Statistics

For all statistical analyses, confirm that the following items are present in the figure legend, table legend, main text, or Methods section.

n/a	Confirmed	
☐	☒	The exact sample size (n) for each experimental group/condition, given as a discrete number and unit of measurement
☐	☒	A statement on whether measurements were taken from distinct samples or whether the same sample was measured repeatedly
☐	☒	The statistical test(s) used AND whether they are one- or two-sided<br>*Only common tests should be described solely by name; describe more complex techniques in the Methods section.*
☐	☒	A description of all covariates tested
☐	☒	A description of any assumptions or corrections, such as tests of normality and adjustment for multiple comparisons
☐	☒	A full description of the statistical parameters including central tendency (e.g. means) or other basic estimates (e.g. regression coefficient) AND variation (e.g. standard deviation) or associated estimates of uncertainty (e.g. confidence intervals)
☐	☒	For null hypothesis testing, the test statistic (e.g. F, t, r) with confidence intervals, effect sizes, degrees of freedom and P value noted<br>*Give P values as exact values whenever suitable.*
☒	☐	For Bayesian analysis, information on the choice of priors and Markov chain Monte Carlo settings
☒	☐	For hierarchical and complex designs, identification of the appropriate level for tests and full reporting of outcomes
☐	☒	Estimates of effect sizes (e.g. Cohen's d, Pearson's r), indicating how they were calculated

Our web collection on statistics for biologists contains articles on many of the points above.

Software and code

Policy information about availability of computer code

Data collection	No software was used.
Data analysis	Canu ver. 1.4, RepeatScout ver. 1.0.5, RepeatMasker ver. 4.1.1, HaploMerger2 ver. 20180603, Juicer ver. 1.6, 3D-DNA pipeline ver. 180114, Juicebox v1.11.08, R ver. 4.0.3, PPCAC annotation pipeline ver. 1.0, lastz ver. 1.04.00, salmon ver. 1.1.0, BWA ver. 0.7.17, samtools ver. 1.9, bcftools ver. 1.8-12. HISAT2 ver. 2.2.1, MUSCLE ver. 3.8.31, PAML ver. 4.9j. Custom scripts used in the analysis are avilable from https://github.com/KYPpa/Ppac_Pexs_Spec/tree/master.

For manuscripts utilizing custom algorithms or software that are central to the research but not yet described in published literature, software must be made available to editors and reviewers. We strongly encourage code deposition in a community repository (e.g. GitHub). See the Nature Portfolio guidelines for submitting code & software for further information.

Data

Policy information about availability of data

All manuscripts must include a data availability statement. This statement should provide the following information, where applicable:

- Accession codes, unique identifiers, or web links for publicly available datasets
- A description of any restrictions on data availability
- For clinical datasets or third party data, please ensure that the statement adheres to our policy

Data used in the analyses are available in the figshare under https://figshare.com/projects/Dataset_of_speciation_genetics_of_P_pacificus_vs_P_exspectatus/132506 at time of publication.

Field-specific reporting

Please select the one below that is the best fit for your research. If you are not sure, read the appropriate sections before making your selection.

☐ Life sciences ☐ Behavioural & social sciences ☒ Ecological, evolutionary & environmental sciences

For a reference copy of the document with all sections, see nature.com/documents/nr-reporting-summary-flat.pdf

Ecological, evolutionary & environmental sciences study design

All studies must disclose on these points even when the disclosure is negative.

Study description	Crossing experiments, genome assembly, cytogenetics, recombination rate analysis, QTL analysis, and genome analysis with re-sequencing data
Research sample	Collection of worms from culture. Sampling of hybrid individuals after crosses between different strains for genotype.
Sampling strategy	Sampling of all individuals of the target generation and sex that can be obtained in a single experiment
Data collection	Phenotyping of hybrids with crossing experiments. Whole genome sequencing using single-molecule, HiC and short-read sequencing. Cytogenetic analysis. Collection of the reference data via public repositories.
Timing and spatial scale	We selected the earliest generation for QTL analysis. The later generations makes the interpretation difficult. Different cross experiments were done in different time. The results are consistently supporting for the same conclusion.
Data exclusions	No data was excluded.
Reproducibility	Different experiments are consistently supporting for the same conclusion.
Randomization	Samples were not allocated to different groups. Phenotyping of single individual was done in isolation.
Blinding	No blinding

Did the study involve field work? ☐ Yes ☒ No

Reporting for specific materials, systems and methods

We require information from authors about some types of materials, experimental systems and methods used in many studies. Here, indicate whether each material, system or method listed is relevant to your study. If you are not sure if a list item applies to your research, read the appropriate section before selecting a response.

Materials & experimental systems

n/a	Involved in the study	
☒	☐	Antibodies
☒	☐	Eukaryotic cell lines
☒	☐	Palaeontology and archaeology
☐	☒	Animals and other organisms
☒	☐	Human research participants
☒	☐	Clinical data
☒	☐	Dual use research of concern

Methods

n/a	Involved in the study	
☒	☐	ChIP-seq
☒	☐	Flow cytometry
☒	☐	MRI-based neuroimaging

Animals and other organisms

Policy information about studies involving animals; ARRIVE guidelines recommended for reporting animal research

Laboratory animals	Pristionchus pacificus, PS312, RS3066, RS5221, RSA635, RSB001; Pristionchus exspectatus, RS5522, RS5522B; Pristionchus occultus, RS5811, RS5811B1, RS5915, RS5920, RS5930; Pristionchus sikae, RS5901; Pristionchus arcanus, RS5527; Pristionchus kurosawai, RS5914; Pristionchus taiwanensis RS5797; Pristionchus maxplancki, RS5594. Different sexes and ages are used depending on the experiments.
Wild animals	No wild animals
Field-collected samples	No field-collected sampels

Ethics oversight	Nematodes are not included in the animal welfare act of Germany. No ethical approval or guidance was required.

Note that full information on the approval of the study protocol must also be provided in the manuscript.
